# Mice learn to avoid regret

**DOI:** 10.1371/journal.pbio.2005853

**Published:** 2018-06-21

**Authors:** Brian M. Sweis, Mark J. Thomas, A. David Redish

**Affiliations:** 1 Graduate Program in Neuroscience & Medical Scientist Training Program, University of Minnesota, Minneapolis, Minnesota, United States of America; 2 Department of Neuroscience, University of Minnesota, Minneapolis, Minnesota, United States of America; 3 Department of Psychology, University of Minnesota, Minneapolis, Minnesota, United States of America; University of Oxford, United Kingdom of Great Britain and Northern Ireland

## Abstract

Regret can be defined as the subjective experience of recognizing that one has made a mistake and that a better alternative could have been selected. The experience of regret is thought to carry negative utility. This typically takes two distinct forms: augmenting immediate postregret valuations to make up for losses, and augmenting long-term changes in decision-making strategies to avoid future instances of regret altogether. While the short-term changes in valuation have been studied in human psychology, economics, neuroscience, and even recently in nonhuman-primate and rodent neurophysiology, the latter long-term process has received far less attention, with no reports of regret avoidance in nonhuman decision-making paradigms. We trained 31 mice in a novel variant of the Restaurant Row economic decision-making task, in which mice make decisions of whether to spend time from a limited budget to achieve food rewards of varying costs (delays). Importantly, we tested mice longitudinally for 70 consecutive days, during which the task provided their only source of food. Thus, decision strategies were interdependent across both trials and days. We separated principal commitment decisions from secondary reevaluation decisions across space and time and found evidence for regret-like behaviors following change-of-mind decisions that corrected prior economically disadvantageous choices. Immediately following change-of-mind events, subsequent decisions appeared to make up for lost effort by altering willingness to wait, decision speed, and pellet consumption speed, consistent with past reports of regret in rodents. As mice were exposed to an increasingly reward-scarce environment, we found they adapted and refined distinct economic decision-making strategies over the course of weeks to maximize reinforcement rate. However, we also found that even without changes in reinforcement rate, mice transitioned from an early strategy rooted in foraging to a strategy rooted in deliberation and planning that prevented future regret-inducing change-of-mind episodes from occurring. These data suggest that mice are learning to avoid future regret, independent of and separate from reinforcement rate maximization.

## Author summary

Regret describes a unique postdecision phenomenon in which losses are realized as a fault of one’s own actions. Regret is often hypothesized to have an inherent negative utility, and humans will often incur costs so as to avoid the risk of future regret. However, current models of nonhuman decision-making are based on reward maximization hypotheses. We recently found that rats express regret behaviorally and neurophysiologically on neuroeconomic foraging tasks; however, it remains unknown whether nonhuman animals will change strategies so as to avoid regret, even in the absence of changes in the achieved rate of reinforcement. Here, we provide the first evidence that mice change strategies to avoid future regret, independent of and separate from reinforcement rate maximization. Our data suggest mice accomplish this by shifting from a foraging decision-making strategy that produces change-of-mind decisions after investment mistakes to one rooted in deliberation that learns to plan ahead.

## Introduction

Regretful experiences comprise those in which an individual recognizes a better decision could have been made in the past. Humans assert a strong desire to avoid feeling regret [1]. Regret can have an immediate impact on influencing subsequent valuations, but it can also motivate individuals to learn to avoid future regret-provoking scenarios altogether [2]. Recently, the experience of regret has been demonstrated in nonhuman animals, sharing principal neurophysiological and behavioral correlates of regret with humans [3–4]. However, it remains unclear if nonhuman animals are capable of learning from regret in order to avoid recurring episodes in the future.

Counterfactual reasoning, or considering what might have been, is a critical tenet of experiencing regret [5–6]. This entails reflecting on potentially better alternatives that could have been selected in place of a recent decision. Thus, owning a sense of choice responsibility and acknowledging error of one’s own agency is central to regret. Following the experience of regret, humans often report a change in mood and augment subsequent decisions in an attempt at self-justification or in efforts to make up for their losses [7–8]. These immediate effects of regret on behavior describe a phenomenon distinct from the notion that individuals will also learn to take longitudinal measures to avoid future scenarios that may induce regret.

Neuroeconomic decision-making tasks offer a controlled laboratory approach to operationalize and characterize decision-making processes comparable across species [9–12]. Recently, a study by Steiner and Redish reported the first evidence of regret in rodents tested on a spatial decision-making task (Restaurant Row) [4]. In this task, food-restricted rats were trained to spend a limited time budget earning food rewards of varying costs (delays) and demonstrated stable subjective valuation policies of willingness to wait contingent upon cued offer costs. In rare instances in which rats disadvantageously violated their decision policies and skipped low-cost offers only to discover worse offers on subsequent trials (e.g., made “economic mistakes”), they looked back at the previous reward site and displayed corrective decisions that made up for lost time. These behaviors coincided with neural representations of retrospective missed opportunities in the orbitofrontal cortex, consistent with human and nonhuman-primate reports of counterfactual “might-have-been” representations [2–4,8,13–15]. While these data demonstrate that rats are responsive to the immediate effects of regret, the regret instances were too sparse to determine whether rats also showed long-term consequences of these regret phenomena. Thus, it remains unknown if nonhuman animals are capable of learning from such regret-related experiences, leaving open the question of whether nonhuman animals adopt longitudinal changes in economic decision-making strategies that prevent future instances of regret from occurring in the first place.

**Fig 1:**
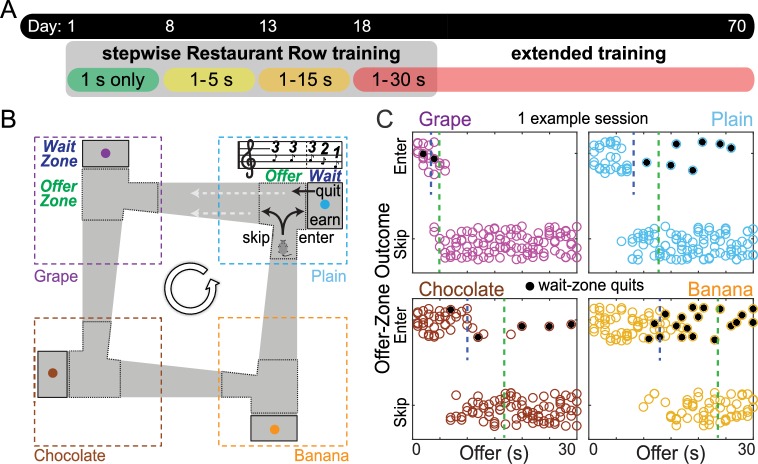
Longitudinal economic design of the Restaurant Row task. (A) Experimental timeline. Mice were trained for 70 consecutive d, earning their only source of food on this task. Stages of training were broken up into blocks in which the range of possible offers began in a reward-rich environment (all offers were always 1 s, green epoch) and escalated to increasingly reward-scarce environments (offer ranges of 1–5 s, 1–15 s, 1–30 s). (B) Task schematic. Food-restricted mice were trained to encounter serial offers for flavored rewards in 4 “restaurants.” Restaurant flavor and location were fixed and signaled via contextual cues. Each restaurant contained a separate offer zone and wait zone. Tones sounded in the offer zone; fixed tone pitch indicated delay (randomly selected from that block’s offer range) mice would have to wait in the wait zone. Tone pitch descended during delay “countdown” if mice chose to enter the wait zone. Mice could quit the wait zone for the next restaurant during the countdown, terminating the trial. Mice were tested daily for 60 min. (C) Example session (from the 1–30 s red epoch) with individual trials plotted as dots. This representative mouse entered low delays and skipped high delays in the offer zone while sometimes quitting once in the wait zone (black dots). Dashed vertical lines represent calculated offer zone (green) and wait zone (blue) “thresholds” of willingness to budget time. Thresholds were measured from the inflection point of fitting a sigmoid curve to enters versus skips or earns versus quits as a function of delay cost. Data available as a supplemental file.

In the present study (Fig 1), we trained food-restricted mice to traverse a square maze with 4 feeding sites (restaurants), each with unique spatial cues and providing a different flavor (Fig 1B). On entry into each restaurant, mice were informed of the delay that they would be required to wait to get the food from that restaurant. In this novel variant of the Restaurant Row task, each restaurant contained 2 distinct zones: an offer zone and a wait zone. Mice were informed of the delay on entry into the offer zone, but delay countdowns did not begin until mice moved into the wait zone. Thus, in the offer zone, mice could either enter the wait zone (to wait out the delay) or skip (to proceed on to the next restaurant). After making an initial enter decision, mice had the opportunity to make a secondary reevaluative decision to abandon the wait zone (quit) during delay countdowns (S1 Video). Just like rats, mice revealed preferences for different flavors that varied between animals but were stable across days, indicating subjective valuations for each flavor were used to guide motivated behaviors. Varying flavors, as opposed to varying pellet number, allowed us to manipulate reward value without introducing differences in feeding times between restaurants (as time is a limited commodity on this task). Costs were measured as different delays mice would have to wait to earn a food reward on that trial, detracting from their session’s limited 1 h time budget. Delays were randomly selected between a range of offers for each trial. Tones sounded upon restaurant entry whose pitch indicated offer cost and descended in pitch stepwise during countdowns once in the wait zone.

Taken together, in this task, mice must make serial judgements in a self-paced manner, weighing subjective valuations for different flavors against offer costs and balancing the economic utility of sustaining overall food intake against earning more rewards of a desirable flavor. In doing so, cognitive flexibility and self-control become critical components of decision-making valuation processes in this task, assessed in 2 separate stages of decision conflict (in the offer and wait zones). Importantly, because mice had 1 h to work for their sole source of food for the day, trials on this task were interdependent both within and across days. Therefore, this was an economic task in which time must be budgeted in order to become self-sufficient across days. Here, we tested mice for 70 consecutive d. Thus, the key to strategy development on this task is the learning that takes place across days, for instance, when performance on a given day produces poor yield. Monitoring longitudinal changes in decision-making strategy can provide novel insight into regret-related learning experiences.

## Results

**Fig 2:**
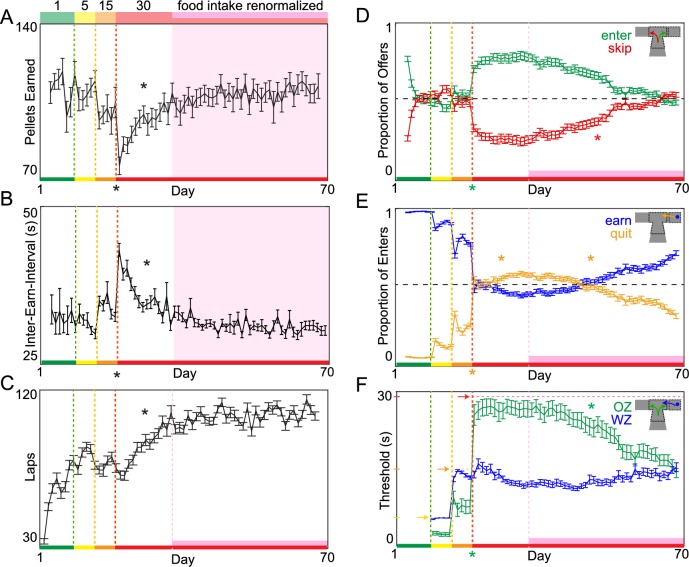
Changes in economic decisions in an increasingly reward-scarce environment. (A-B) Primary dependent variables: total earned food intake (A) and reinforcement rate (B), measured as average time between earnings. Transition to the 1–30 s block caused a significant decrease in food intake and reinforcement rate. By approximately day 32, food intake and reinforcement rate renormalized back to stable baseline levels compared to previous testing in reward-rich environments. The epoch marked in pink defines this renormalization to baseline and is used throughout the remaining longitudinal plots. (C) Number of self-paced laps run (serially encountering an offer in each of the 4 restaurants). (D) Proportion of total offers entered versus skipped. Horizontal dashed line represents 0.5 level. (E) Proportion of total enters earned versus quit. Horizontal dashed line represents 0.5 level. (F) Economic decision thresholds: OZ and WZ choice outcomes as a function of cost. Horizontal dashed lines represent the maximum possible threshold in each block. Data are presented as the cohort’s (*N* = 31) daily means (±1 SE) across the entire experiment. Color code on the x-axis reflects the stages of training (offer cost ranges denoted from 1 to the number on the top of panel A). Vertical dashed lines (except pink) represent offer block transitions. * on the x-axis indicates immediate significant behavioral change at the block transition; otherwise, * indicates gradual significant changes within the 1–30 s block during either the early 2 wk adaptation period or late pink epoch. Data available as a supplemental file. OZ, offer zone; WZ, wait zone

How mice were trained on the Restaurant Row task allowed us to characterize the development of and changes in economic decision-making strategies. Mice progressed from a reward-rich to a reward-scarce environment in blocks of stages of training across days (Fig 1A). Each block was defined by the range of possible costs that could be encountered when offers were randomly selected on the start of each trial upon entry into each restaurant’s offer zone. The first block (green epoch) spanned 7 d in which all offers were always 1 s (Fig 1A). During this time, mice quickly learned the structure of the task (Fig 2), becoming self-sufficient and stabilizing the number of pellets earned (Fig 2A), reinforcement rate (Fig 2B), and number of laps run (Fig 2C). During this block, mice rapidly developed stable flavor preferences and learned to skip offers for less-preferred flavors and enter offers for more-preferred flavors, entering versus skipping at roughly equal rates overall while rarely quitting (Fig 2D and 2E, S1A–S1E Fig). The second block (yellow epoch) spanned 5 d in which offers could range between 1–5 s. The third block (orange epoch) spanned 5 d in which offers could range between 1–15 s. Lastly, the fourth and final block (red epoch, beginning on day 18) lasted until the end of this experiment (day 70), in which offers could range between 1–30 s. Note that because the mice had a limited 1 h time budget to get all of their food for the day, these changes in offer distributions produced increasingly reward-scarce environments that required more complex strategies to maximize rate of reward.

Upon transitioning to the 1−30 s offer block, mice suffered a large drop in total number of pellets earned (Fig 2A, repeated measures ANOVA, *F* = 9.46, *p* < 0.01) and reinforcement rate (increase in time between earnings, Fig 2B, *F* = 253.93, *p* < 0.0001). With this came a number of changes in decision-making behaviors that took place immediately, on an intermediate timescale, and on a delayed long-term timescale. Decreases in food intake and reinforcement rate were driven by an immediate significant increase in proportion of total offers entered (Fig 2D, *F* = 56.10, *p* < 0.0001) coupled with a significant increase in proportion of entered offers quit (Fig 2E, *F* = 472.88, *p* < 0.0001) as mice experienced long delays in the wait zone for the first time. This suggests that mice were apt to accept expensive offers in the offer zone even though they did not actually earn those offers in the wait zone (S2C, S2G, S2I and S2J Fig). This also suggests that choosing to enter versus skip in the offer zone and choosing to opt out of waiting in the wait zone may access separate valuation algorithms. We quantified this disparity in economic valuations by calculating separate “thresholds” of willingness to enter in the offer zone and willingness to wait in the wait zone as a function of offer cost. Following the 1−30 s transition, offer zone thresholds significantly increased (maxed out at approximately 30 s) and became significantly higher than wait zone thresholds (Fig 2F, offer zone change: *F* = 151.65, *p* < 0.0001; offer zone versus wait zone: *F* = 59.85, *p* < 0.0001). Furthermore, we found that these immediate behavioral changes were more robust in more-preferred restaurants, suggesting asymmetries in suboptimal decision-making strategies upon transition from a reward-rich to a reward-scarce environment were dependent on differences in subjective valuation algorithms (S1A Fig, see S1 Text).

Because performance on this task served as the only source of food for these mice, decision-making policies that might have been sufficient in reward-rich environments must change when they are no longer sufficient in reward-scarce environments. We found that mice demonstrated behavioral adaptations over the 2 wk following the transition to the 1−30 s offer range so that by approximately day 32, they had effectively restored overall food intake (Fig 2A, change across 2 wk: *F* = 355.21, *p* < 0.0001; post-2 wk compared to baseline: *F* = 0.80, *p* = 0.37) and reinforcement rates (Fig 2B, change across 2 wk: *F* = 183.68, *p* < 0.0001; post-2 wk compared to baseline: *F* = 0.24, *p* = 0.63) to baseline levels similar to what was observed in a reward-rich environment (Fig 2A and 2B). Note that the restored reinforcement rates renormalization, indicated by the pink epoch in Fig 2, was not imposed by the experimenters but was due to changes in the behavior of the mice under unchanged experimental rules (1−30 s offers). Mice accomplished this by running more laps to compensate for food loss (Fig 2C, *F* = 221.61, *p* < 0.0001) without altering economic decision-making policies. That is, we observed no changes in wait zone thresholds during this 2-wk period (Fig 2F, *F* = 2.57, *p* = 0.11). By entering the majority of offers indiscriminately with respect to cost (Fig 2D, proportion trials entered > 0.5: *t* = 31.22, *p* < 0.0001, S2C Fig), mice found themselves sampling more offers in the wait zone they were also unwilling to wait for, leading to an increase in quitting (Fig 2E, *F* = 55.37, *p* < 0.0001, S2G Fig).

**Fig 3:**
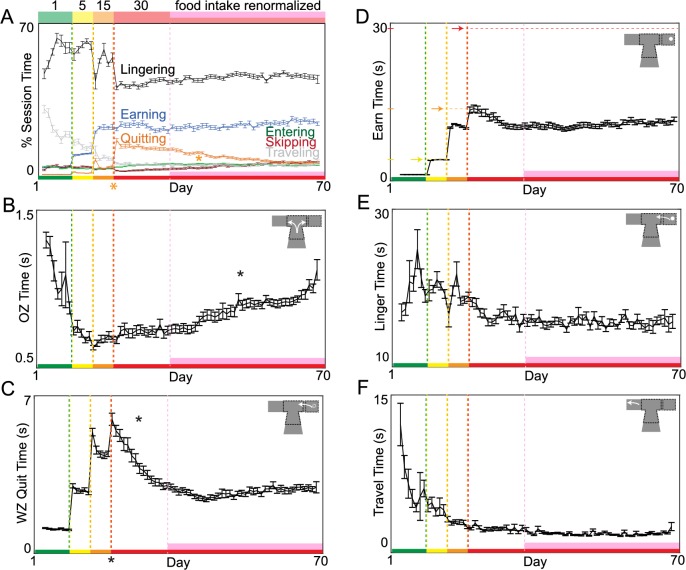
Allocation of a limited time budget among separable decision processes. (A) Cumulative time spent engaged in various separable behaviors and decision processes calculated as percent of the total 1 h daily session’s time budget. (B) Average time in the OZ from offer onset upon restaurant entry until either a skip or enter decision was made. (C) Average time in the WZ from countdown onset until a quit decision was made. (D) Average time in the WZ from countdown onset until a pellet was earned. (E) Average time near the reward site from pellet delivery until mice exited the WZ and entered the hallway, advancing to the next restaurant. (F) Average time spent traveling in the hallway between restaurants between trials (from either a skip, quit, or postearn leave decision until the next trial’s offer onset upon subsequent restaurant entry). Data are presented as the cohort’s (*N* = 31) daily means (±1 SE) across the entire experiment. Color code on the x-axis reflects the stages of training (offer cost ranges denoted from 1 to the number on the top of panel A). Vertical dashed lines (except pink) represent block transitions. * on the x-axis indicates immediate significant behavioral change at the block transition; otherwise, * indicates gradual significant changes within the 1−30 s block during either the early 2 wk adaptation period or late pink epoch. Data available as a supplemental file. OZ, offer zone; WZ, wait zone.

**Fig 4:**
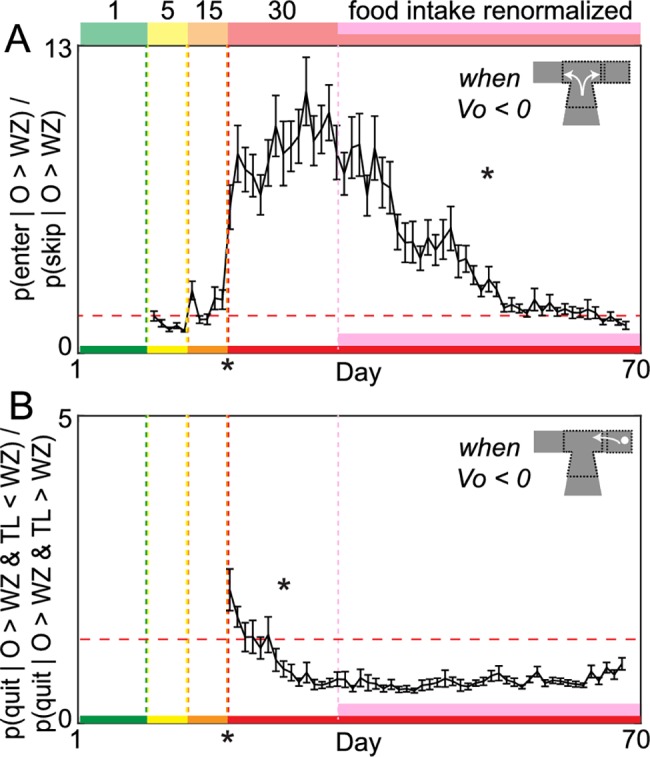
Development of separate intermediate wait zone and long-term offer zone efficient decision-making strategies. (A) Offer zone inefficiency ratio. V_O_ = WZ–O. Probability of entering negatively valued offers relative to the probability of skipping negatively valued offers. Horizontal dashed line indicates equivalent 1:1 ratio of entering versus skipping negatively valued offers. (B) Wait zone inefficiency ratio. V_L_ = WZ–TL. Probability of quitting negatively valued offers when V_L_ was positive relative to when V_L_ was still negative. Horizontal dashed line indicates equivalent 1:1 ratio of quitting inefficiently versus efficiently. Data are presented as the cohort’s (*N* = 31) daily means (±1 SE) across the entire experiment. Color code on the x-axis reflects the stages of training (offer cost ranges denoted from 1 to the number on the top of panel A). Vertical dashed lines (except pink) represent block transitions. * on the x-axis indicates ratio significantly greater than 1:1 immediately following the 1–30 s block transition; otherwise, * indicates gradual significant changes within the 1–30 s block during either the early 2 wk adaptation period or late pink epoch. Data available as a supplemental file. O, offer cost; TL, countdown time left; V_L_, value of time left in countdown at the moment of quitting; V_O_, offer value; WZ, wait zone threshold.

Investing a greater portion of a limited time budget waiting for rewards that are ultimately abandoned appears, at face value, to be a wasteful decision-making strategy. Yet mice were able to restore food intake and reinforcement rates using this strategy. We characterized how mice allocated their limited time budget and quantified time spent among various separable behaviors that made up the total 1-h session (Fig 3). We first calculated the percent of total budget engaged in making offer zone decisions to skip versus enter, wait zone decisions to quit versus earn, postearn consumption behaviors, and travel time between restaurants (Fig 3A). We also calculated the average time spent engaged in a single bout of each decision process (Fig 3B–3F). The percent of total session time allocated to quit events (Fig 3A, *F* = 306.72, *p* < 0.0001), as well as average time spent waiting before quitting (Fig 3C, *F* = 44.21, *p* < 0.0001), significantly increased immediately following the transition to 1−30 s offers. Thus, time spent waiting in the wait zone before engaging in change-of-mind behaviors drove the immediate decrease in reinforcement rates and overall loss of food intake. Note that this waiting and then quitting behavior entails investing time that provided no reward. Over the subsequent 2 wk, time spent waiting before quitting significantly decreased as mice restored food intake and reinforcement rates (Fig 3C, *F* = 781.55, *p* < 0.0001). This suggests that mice learned to quit more efficiently in the wait zone. We calculated economic efficiency of wait zone quits (Fig 4B) by measuring how much time was remaining in the countdown at the moment of quitting relative to an individual’s wait zone threshold. Over these 2 wk, mice learned to quit in a more economically advantageous manner before excess time was invested. That is, mice learned to quit while the time remaining in the countdown was still above wait zone thresholds (Fig 4B, *F* = 64.00, *p* < 0.0001, S1P Fig, S3 Fig, see S1 Text), avoiding quitting at a timepoint when it would have been advantageous to otherwise finish waiting. This suggests that wait zone–quit reevaluations were corrective actions that opposed erroneous principal valuations in the offer zone. Interestingly, mice struggled to learn to quit efficiently in more preferred restaurants, reflecting a reluctance to apply adaptive opt-out foraging strategies in situations with high subjective valuation biases (S1K and S1P Fig see S1 Text). Despite increasing change-of-mind efficiency, because the frequency of quit events increased along this 2 wk time course, the fraction of the session budget allocated to quit events remained significantly elevated compared to baseline (Fig 3A, *F* = 105.90, *p* < 0.0001).

After mice successfully restored food intake and reinforcement rates by refining a foraging strategy, we found a distinct, delayed phase of additional learning that took place with prolonged training in the absence of any further changes in food intake (pink epoch, Fig 2A, *F* = 1.82, *p* = 0.18), reinforcement rates (pink epoch, Fig 2B, *F* = 0.01, *p* = 0.95), or laps run (pink epoch, Fig 2C, *F* = 1.54, *p* = 0.21). The proportion of enter-then-quit decisions decreased over the remainder of the experiment (Fig 2E, *F* = 159.30, *p* < 0.0001) as mice learned to reject offers in the offer zone that they were unwilling to remain committed to once in the wait zone (S2D–S2H Fig). This is reflected in a decrease in offer zone thresholds until they were in register with wait zone thresholds by the end of the experiment (pink epoch, Fig 2F, offer zone change: *F* = 812.40, *p* < 0.0001; offer zone versus wait zone at day 70: *F* = 0.17, *p* = 0.68). As a result, mice learned to skip more often in the offer zone (pink epoch, Fig 2D, *F* = 116.85, *p* < 0.0001). We calculated the economic efficiency of offer zone decisions by measuring the likelihood of skipping offers above wait zone thresholds relative to the likelihood of entering offers above wait zone threshold and found that offer zone decisions became more efficient during the pink epoch (Fig 4A, *F* = 474.94, *p* < 0.0001). As a result, the proportion of session budget allocated to quit events declined back to baseline levels (pink epoch, Fig 3A, budget quitting change: *F* = 1639.61, *p* < 0.0001, day 70 compared to baseline: *F* = 0.17, *p* = 0.68). The only change observed in average time spent per decision across decision processes during this phase of learning was in offer zone time, which increased over extended training as skip frequency increased (pink epoch, Fig 3B, offer zone time: *F* = 490.14, *p* < 0.0001; wait zone quit time: *F* = 0.10, *p* = 0.75; earn time: *F* = 0.11, *p* = 0.74; linger time: *F* = 0.73, *p* = 0.39; travel time: *F* = 0.01, *p* = 0.94).

**Fig 5:**
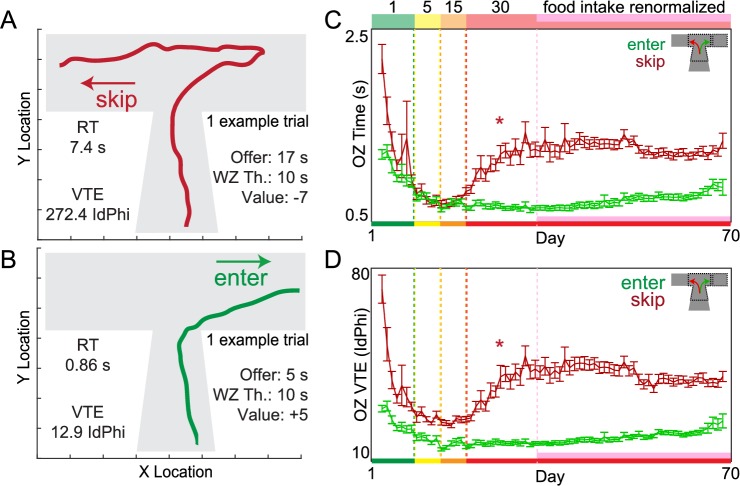
Development of deliberative behaviors during principal OZ valuations. (A-B) Example **x** and **y** locations of a mouse’s path trajectory in the OZ (wait zone not depicted) over time during a single trial (from day 70). (A) Skip decision for a high-delay offer. The mouse initially oriented toward entering (right) but then ultimately reoriented to skip (left). WZ Th. minus offer captures the relative subjective “value” of the offer. Negative value denotes an economically unfavorable offer. (B) Enter decision for positively valued offer; rapid without reorientations. This OZ trajectory pattern is indistinguishable from enter-then-quit decisions for negatively valued offers. (C) Average OZ RT split by enter versus skip decisions across days of training. (D) Average OZ VTE behavior split by enter versus skip decisions across days of training. Data are presented as the cohort’s (*N* = 31) daily means (±1 SE) across the entire experiment. Color code on the x-axis in (C-D) reflects the stages of training (offer cost ranges denoted from 1 to the number on the top of panel C). Vertical dashed lines (except pink) represent block transitions. * indicates gradual significant changes within the 1–30 s block during the early 2 wk adaptation period. Data available as a supplemental file. OZ, offer zone; RT, reaction time; VTE, vicarious trial and error; WZ Th., wait zone threshold.

Upon closer examination of offer zone behaviors (Fig 5), we found marked changes following the 1–30 s transition in skip decisions but not in enter decisions. We calculated the reaction time from offer onset until either a skip or enter decision was made. We also tracked each animal’s ***x*** and ***y*** location path trajectory as they passed through the offer zone. From this, we could capture the degree to which animals interrupted smooth offer zone passes with “pause and look” reorientation behaviors known as vicarious trial and error (VTE). VTE is a well-studied behavioral phenomenon that reveals ongoing deliberation and planning during moments of embodied indecision, supported by numerous electrophysiological experiments reporting concurrent neural representations of possible future outcomes compared serially [16–25]. The physical “hemming and hawing” characteristic of VTE is best measured by calculating changes in velocity vectors of discrete body ***x*** and ***y*** positions over time as ***dx*** and ***dy***. From this, we can calculate the momentary change in angle, *Phi*, as *dPhi*. When this metric is integrated over the duration of the pass through the offer zone, VTE is measured as the absolute integrated angular velocity, or *IdPhi*, until either a skip or enter decision was made (Fig 5A and 5B, day 70 examples path traces). In a reward-rich environment, offer zone reaction time became more rapid (green-yellow-orange epochs, Fig 5C, *F* = 157.78, *p* < 0.0001), and paths measured by *IdPhi* became more stereotyped (green-yellow-orange epochs, Fig 5D, *F* = 150.19, *p* < 0.0001) as mice learned the structure of the task and made ballistic decisions. However, in a reward-scarce environment, skip reaction time (Fig 5C, *F* = 92.00, *p* < 0.0001) and skip VTE (Fig 5D, *F* = 117.80, *p* < 0.0001) began to increase following the transition to 1–30 s offers. These behaviors stabilized after food intake, and reinforcement rates were restored for the remainder of the experiment (pink epoch, Fig 5C, skip time: *F* = 2.21, *p* = 0.14; Fig 5D, skip VTE: *F* = 0.45, *p* = 0.50) as offer zone thresholds declined (Fig 2F) and skip frequency increased (Fig 2D). This suggests that mice enacted deliberative strategies in the offer zone after prolonged training. Mice learned to plan to skip expensive offers that previously would have been rapidly entered and then ultimately quit. Furthermore, following the transition to 1–30 s offers, enter decisions remained fast (Fig 5C, *F* = 1.73, *p* = 0.19) with low VTE (Fig 5D, *F* = 0.97, *p* = 0.32), suggesting enter decisions that ultimately led to quits were economically disadvantageous snap judgements in the offer zone that were subsequently reevaluated and corrected in the wait zone. Skip reaction time and VTE were higher in more preferred restaurants (S1G–S1J Fig), suggesting decisions to skip expensive offers for desired flavors were more difficult. Furthermore, refining the economic efficiency of this deliberative strategy was more difficult to learn in more-preferred restaurants (S1O Fig, S4 Fig, S5 Fig, see S1 Text).

This opens an intriguing question: if the changes that took place with prolonged training did not change the efficiency of food receipt, and if the only change after the development of deliberative strategies was a reversal of the increase in quit frequency, what does a reduction in change-of-mind decisions serve these animals? Given that there was no gain in food intake or reinforcement rate nor decrease in energy expenditure, what might be the driving force behind this delayed learning process?

A strength of the Restaurant Row task is its capability of measuring how economic decisions in one trial influence economic decisions in the following trial. This between-trial sequence feature of Restaurant Row captures post-decision-making phenomena, like regret [4]. A key factor in experiencing regret is the realization that a user-driven mistake has been made and that an alternative response could have led to a more ideal outcome. A change-of-mind quit decision in this novel variant of the Restaurant Row task thus presents an economic scenario in which mice take action to opt out of and abandon ongoing investments in the wait zone following an economically disadvantageous enter decision. As shown above, quits are economically advantageous reevaluations of prior snap judgements made in the offer zone. Thus, quit events reveal a potential economic scenario in which an agent’s decision has led to an economically disadvantageous option, whereby a counterfactual opportunity (“should have skipped it in the first place”) could provoke a regret-like experience.

**Fig 6:**
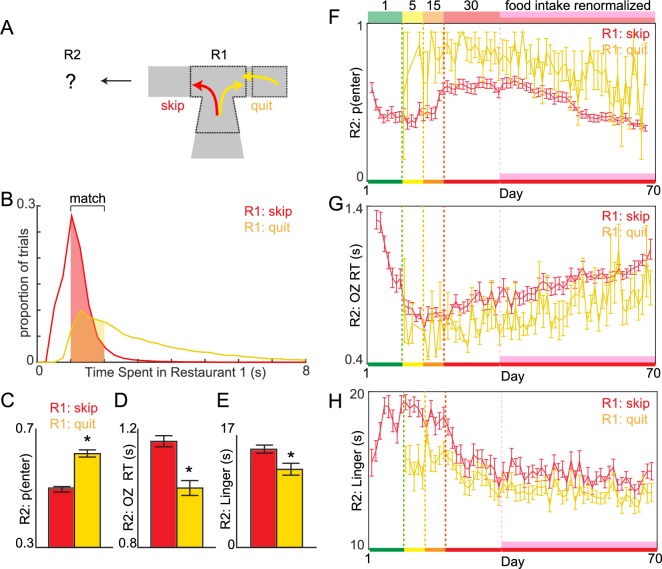
Regret-like sequence effects following change-of-mind wait zone reevaluations. (A) Following either a skip or enter-then-quit decision in R1, we characterized behaviors on the subsequent trial in R2. (B) Distribution of time spent in R1 from offer onset until a skip decision (OZ time) or quit decision (OZ time plus wait zone time) was made. To control for the effects of differences in time spent skipping versus entering-then-quitting in R1 on behavior in Restaurant 2, we compared trials matched for resource depletion between conditions. (C-D) Data averaged across the 1–30 s offer block. (C) Probability of entering an offer in R2 after skipping versus quitting in R1. (D) OZ RT in R2 after skipping versus quitting in R1. (E) Time spent consuming an earned pellet and lingering at the reward site in R2 after skipping versus quitting in R1. (F-H) Postskip versus post-enter-then-quit sequence data across the entire experiment from (C-E), respectively. Data are presented as the cohort’s (*N* = 31) means (±1 SE). Color code on the x-axis in (F-H) reflects the stages of training (offer cost ranges denoted from 1 to the number on the top of panel F). Vertical dashed lines (except pink) represent block transitions. * indicate significant difference between skip versus quit conditions. Data available as a supplemental file. OZ, offer zone; R1, restaurant 1; R2, restaurant 2; RT, reaction time.

Economic theories of human decision-making have hypothesized that regret adds a negative component to a utility function [1,7,26–28]. These theories suggest that an important driving force for human decision-making is the avoidance of future regret [2,8,29–31]. In order to test if decisions following enter-then-quit sequences carry added negative utility akin to regret previously demonstrated in Restaurant Row, we examined decision outcomes in the subsequent restaurant encounter following change-of-mind decisions compared to those following skip decisions (Fig 6). We compared enter-then-quit events to skip events (Fig 6A) that were matched for total time spent in the first restaurant before ultimately turning down the offer and advancing to the subsequent restaurant (Fig 6B). For example, we compared a skip decision that used up 2 s of offer zone time to an enter-then-quit sequence that used up a total of 2 s of combined offer zone and wait zone time. Consistent with previous reports in rats who attempted to make up for lost effort following regret, we found that, following quits, mice were more likely to accept offers in the next trial (Fig 6C, *F* = 39.26, *p* < 0.0001), did so quickly (Fig 6D, *F* = 163.28, *p* < 0.0001), and upon earning subsequent rewards, rapidly consumed food and exited the reward site (Fig 6E *F* = 191.89, *p* < 0.0001), compared to trials following skips. Quit-induced effects on subsequent trials existed across the entire experiment (Fig 6F–6H) and remained, even after controlling for flavor preferences (S6 Fig, see S1 Text). This suggests that enter-then-quit sequences were capable of augmenting subsequent valuations, even when change-of-mind reevaluations were matched to skip decisions for resource depletion and even during early stages of training amidst simpler foraging strategies before deliberative strategies developed.

Taken together, on a multiple-week timescale, mice transitioned from a foraging strategy that learned to become efficient (Fig 4B) to a distinct deliberative strategy that separately learned to become efficient later (Fig 4A). This change in strategy effectively traded enter-then-quit reevaluative decisions in the wait zone for skip decisions during principal valuations in the offer zone, with no overt benefit other than reducing the frequency of change-of-mind events. Quit events and skip events came from the same distribution of offer lengths (S7 Fig). Based on these data, it seems that not only can a change-of-mind experience have an immediate impact on subsequent valuations but it can also impact longer-term learning in mice capable of augmenting decision-making strategies. The resulting decision-making strategy appears to be one rooted in deliberation and planning as a means of avoiding future change-of-mind scenarios altogether.

## Discussion

Numerous studies have demonstrated that human individuals develop long-term strategies to avoid future instances of regret [2,7–8,14]. This phenomenon is distinct from the ability of regret to drive compensatory augmentations in valuation processes of immediately subsequent opportunities. While the immediate effects of regret have been demonstrated in rodents [4], long-term regret-avoidance learning, however, has not been previously observed. Here, we provide support not only for growing evidence that rodents (mice as well as rats) are capable of experiencing regret-like episodes but also that such experiences, separate from and independent of reinforcement maximization, can drive long-term changes in decision-making strategies.

Much of the animal learning literature has focused primarily on reinforcement maximization as the sole motivator of reward-related learning in decision-making paradigms [32–35]. That is, the goal of increasing reward reinforcement rate is thought to underlie animal behavior. Temporal difference error algorithms demonstrate a well-characterized mechanism of reward maximization–driven motivation in reinforcement learning theory [33–36]. Such learning algorithms, supported by neural representations of escalating response vigor and reward expectancies in mesolimbic dopamine systems, update behavioral policies or learn novel contingencies in order to optimize a given cost function and produce maximum reward yield [37–42]. Behavioral and neurophysiological data in both humans and nonhuman animals support a reward maximization theory of learning algorithms.

In the present study, we found evidence of reward-maximization learning algorithms as mice progressed from reward-rich to reward-scarce environments and made increasingly efficient wait zone decisions in a self-paced manner on a time-sensitive economic decision-making task during which they earned their only source of food. We also found distinct learning processes separated across space and time in the offer zone that took place on a much longer timescale. We found that mice reduced the frequency of wait zone change-of-mind decisions by learning to plan ahead in the offer zone, without any additional gain in reinforcement rates or reduction in energy expenditure. Other hypothesized drivers of human learning besides reinforcement maximization and energy expenditure minimization include managing affective states, particularly ameliorating or minimizing negative affect [43–44]. Avoiding pain, stress, threat, or anxiety is a well-studied motivator in human learning as well as in nonhuman-animal fear conditioning or punishment learning paradigms [45–46]. However, in a reward context, negative affect associated with regret and reward-related outcome experiences, while well-characterized in humans, is far less understood in animal learning models of positive reinforcement, reward-seeking learning.

The relatively straightforward view of reward maximization–driven reinforcement learning is challenged by the decision-making phenomena made tractable in these economic decision-making paradigms [33]. Postdecision regret is a well-known example that poses issues for traditional reinforcement learning algorithms dependent on updating stimuli or actions associated with actual experienced reward outcomes [33]. Hypothetical outcomes of forgone alternatives processed during counterfactual thinking that turn out to be better than chosen actions—key in regret—are indeed capable of driving long-term changes in future decision strategies through fictive learning, but it is a process that has been sparsely studied in nonhuman animals [3–7,13–15]. Mapping counterfactual outcomes onto corrective actions that could have been taken aids in the development of new decision strategies aimed to avoid regret in the future, yet this is a poorly understood behavioral and neural process.

Change-of-mind behaviors present unique decision-making scenarios that, when assessed on an economic task, can capture the economic advantageous versus disadvantageous nature of principal valuations and subsequent reevaluative choices. On this novel variant of the Restaurant Row task, we separate principal valuations (offer zone) from reevaluative choices (wait zone) across space and time within a single trial. Furthermore, change-of-mind behaviors present a powerful means of studying counterfactual decision processes [47–49]. In the context of the neuroeconomics of regret, a few questions arise: what drives individuals to change their minds? Which decisions might be economically fallible: the original choice, the delayed reconsideration, neither, or both? Why might individuals be reluctant to change their minds, how is this related to regret, and how might this interact with subjective valuation reinforcement learning algorithms?

Change-of-mind decisions occur every day in the real world, yet there is the general consensus that many individuals find this unpleasant and are often reluctant to do so, even when its utility is apparent [50–53]. Imagine the common scenario of a person in a food court during a 1h lunch break deciding which line to wait in—a direct analogue of what we test here in the Restaurant Row task. The decision to back out of waiting in any given line often comes with a sore feeling, even if doing so was an advantageous decision. Conversely, “going down with the ship” describes the sometimes-irrational motivation to refuse overturning a principal judgement and abandoning a partial investment. This is thought to be motivated by a desire to avoid being wasteful, admitting mistakes, or challenging one’s own beliefs. Thus, following an investment history, it is reasonable to appreciate that progress made toward a goal may be difficult to abandon, doing so may generate a source of cognitive dissonance, and thus, the decision to override a principal judgement when reevaluating continued investment errs on the side of perseveration, however economically irrational that may be. This describes a well-known decision-making phenomenon termed the sunk cost fallacy, in which the value of continued investment toward reward receipt is inflated as a function of irrecoverable past investments [54]. Mice, rats, and humans all demonstrate sensitivity to sunk costs in the wait zone when making quit decisions as a function of investment history on translated variants of the Restaurant Row task [55]. Thus, quit-induced regret and sunk cost–driven perseveration appear to be intimately related here. That is, after making a principal judgement in the offer zone to accept an offer at a cost higher than subjective value indicates one should (i.e., an initial economic violation of wait zone threshold), subjects are faced with a change-of-mind dilemma, torn between irrationally waiting out the expensive offer versus rationally backtracking and changing their plans, when affective contributions appear to weigh these options against one another.

In our food court example, the economically rational decision would be to select a line immediately and to make one’s decision while waiting in line. However, this is not what is typically observed—instead, it is far more common for people to deliberate before choosing and investing in any one option, despite the fact that this wastes time planning. Despite reevaluating an ongoing investment being the economically efficient and rational strategy, this hinges on a high frequency of change-of-mind decisions. After prolonged training in the Restaurant Row task, mice show a shift from the select-and-reevaluate foraging strategy to the deliberate-first strategy, even though it produces no change in reinforcement rate or energy expenditure. Thus, we conclude that mice are capable of learning from regret-related experiences induced by change-of-mind decisions and that they develop a forward-looking deliberative strategy that, although expensive in time and in computational resources, is economically advantageous because regret itself induces a negative utility. Rather than learning to deal with regret, sometimes mice take the time to plan ahead and learn to just avoid regret altogether.

## Materials and methods

### Mice

31-C57BL/J6 male mice, 13 wk old, were trained in Restaurant Row. Mice were single-housed (beginning at 11 wk of age) in a temperature- and humidity-controlled environment with a 12 h light/12 h dark cycle with water ad libitum. Mice were food restricted to a maximum of 85% free-feeding body weight and trained to earn their entire day’s food ration during their 1 h Restaurant Row session. Experiments were approved by the University of Minnesota Institutional Animal Care and Use Committee (IACUC; protocol number 1412A-32172) and adhered to NIH guidelines. Mice were tested at the same time every day during their light phase in a dimly lit room, were weighed before and after every testing session, and were fed a small postsession ration in a separate waiting chamber on rare occasions to prevent extremely low weights according to IACUC standards (not <85% free-feeding weights). Previous studies using this task yielded reliable behavioral findings with minimal variability in at least sample sizes of *n* = 7.

### Pellet training

Mice underwent 1 wk of pellet training prior to the start of being introduced to the Restaurant Row maze. During this period, mice were taken off of regular rodent chow and introduced to a single daily serving of BioServ full-nutrition 20 mg dustless precision pellets in excess (5 g). This serving consisted of a mixture of chocolate-, banana-, grape-, and plain-flavored pellets. Next, mice (hungry, before being fed their daily ration) were introduced to the Restaurant Row maze 1 d prior to the start of training and were allowed to roam freely for 15 min to explore, get comfortable with the maze, and familiarize themselves with the feeding sites. Restaurants were marked with unique spatial cues. Restaurant location remained fixed throughout the entire experiment. Feeding bowls in each restaurant were filled with excess food on this introduction day.

### Restaurant Row training

Task training was broken into 4 stages. Each daily session lasted for 1 h. At test start, one restaurant was randomly selected to be the starting restaurant where an offer was made if mice entered that restaurant’s T-shaped offer zone from the appropriate direction in a counterclockwise manner. During the first stage (days 1–7), mice were trained for 1 wk being given only 1 s offers. Brief low-pitch tones (4,000 Hz, 500 ms) sounded upon entry into the offer zone and repeated every second until mice skipped or until mice entered the wait zone, after which a pellet was dispensed. To discourage mice from leaving earned pellets uneaten, motorized feeding bowls cleared an uneaten pellet upon restaurant exit. Leftover pellets were counted after each session, and mice quickly learned to not leave the reward site without consuming earned pellets. The next restaurant in the counterclockwise sequence was always and only the next available restaurant where an offer could be made, such that mice learned to run laps encountering offers across all 4 restaurants in a fixed order serially in a single lap. During the second stage (day 8–12), mice were given offers that ranged from 1 s to 5 s (4,000 Hz to 5,548 Hz, in 387 Hz steps) for 5 d. Offers were pseudorandomly selected, such that all 5 offer lengths were encountered in 5 consecutive trials before being reshuffled, selected independently between restaurants. Again, offer tones repeated every second in the offer zone indefinitely until either a skip or enter decision was made. In this stage and subsequent stages, in the wait zone, 500 ms tones descended in pitch every second by 387 Hz steps, counting down to pellet delivery. If the wait zone was exited at any point during the countdown, the tone ceased, and the trial ended, forcing mice to proceed to the next restaurant. Stage 3 (days 13–17) consisted of offers from 1 s to 15 s (4,000–9,418 Hz) for another 5 d. Stage 4 (days 18–70) offers ranged from 1 s to 30 s (4,000–15,223 Hz) and lasted until mice showed stable economic behaviors. We used 4 Audiotek tweeters positioned next to each restaurant, powered by Lepy amplifiers, to play local tones at 70 dB in each restaurant. We recorded speaker quality to verify frequency playback fidelity. We used Med Associates 20 mg feeder pellet dispensers and 3D-printed feeding bowl receptacles fashioned with mini-servos to control automated clearance of uneaten pellets. Animal tracking, task programming, and maze operation were powered by AnyMaze (Stoelting). Mice were tested at the same time every day in a dimly lit room, were weighed before and after every testing session, and were fed a small postsession ration in a separate waiting chamber on rare occasions as needed to prevent extremely low weights according to IACUC standards (not <85% free-feeding weights).

### Statistical analysis

All data were processed in Matlab, and statistical analyses were carried out using JMP Pro 13 Statistical Discovery software package from SAS. All data are expressed as mean +/− 1 SE. Sample size is included in each figure. No data were lost to outliers. Offer zone thresholds were calculated by fitting a sigmoid function to offer zone choice outcome (skip versus enter) as a function offer length for all trials in a single restaurant for a single session and measuring the inflection point. Wait zone thresholds were calculated by fitting a sigmoid function to wait zone choice outcomes (quit versus earn) as a function of offer length for all entered trials in a single restaurant for a single session. For dynamic analyses that depend on thresholds (e.g., Fig 4), analyses at each timepoint used that timepoint’s threshold information. Statistical significance was assessed using Student *t* tests, one-way, two-way, and repeated-measures ANOVAs, using mouse as a random effect in a mixed model, with post-hoc Tukey *t* tests correcting for multiple comparisons. Significance testing of immediate changes at block transitions were tested using a repeated-measures ANOVA between 1 d pre- and 1 d posttransition. These are indicated by significance annotations below the x-axis on relevant figures. Significance testing of gradual changes within block were tested using a repeated-measures ANOVA across all days within a given block or epoch. These are indicated by significance annotations within the plot either directly above or below the data centered within the epoch of interest. If significant interactions between factors were found (e.g., x rank), these are reflected by multiple significance annotations either below the x-axis or within the plot, respectively. The period of renormalization was estimated based on animal self-driven performance improvements in the 1–30 s block and not imposed on the animals by experimenters nor the protocol design. Renormalization was characterized by identifying the number of days in the 1–30 s block, after which total pellet earnings and reinforcement rate reliably stabilized (within a sliding 5 d window) and was no different from performance in relatively reward-rich environments collapsing across the first 3 training blocks. This was estimated to be approximately by day 30 of the experiment.

## Supporting information

S1 Fig Interactions between subjective flavor preferences and longitudinal economic decision processes. Flavors were ranked from least preferred to most preferred based on total pellet earnings in each restaurant at the end of each session. (A) Pellets earned in each restaurant show early development of flavor preferences that persist throughout the entire experiment. (B) Percentage of offers entered. Horizontal dashed line indicates 100%. (C-E) Total number of trials entered (C), skipped (D), and quit (E). (F) Percentage of entered offers quit. Horizontal dashed line indicates 50%. (G-J) Offer zone behaviors for enter (G, time; I, VTE) and skip (H, time; J, VTE) decisions. (K) Time spent in the wait zone during tone countdown before quitting. (L) Time spent in the wait zone consuming an earned food pellet and lingering near the reward site before advancing to the next trial. (M-N) Offer zone (M) and wait zone (N) thresholds. Horizontal dashed lines represent the maximum possible threshold in each block. (O) Offer zone inefficiency ratio. V_O_ = WZ–O. Probability of entering negatively valued offers relative to the probability of skipping negatively valued offers. Horizontal dashed line indicates equivalent 1:1 ratio of entering versus skipping negatively valued offers. (P) Wait zone inefficiency ratio. V_L_ = WZ–TL. Probability of quitting negatively valued offers when V_L_ was positive relative to when V_L_ was still negative. Horizontal dashed line indicates equivalent 1:1 ratio of quitting inefficiently versus efficiently. (Q) Reward-earning optimality. Proportion of pellets mice actually earned in each restaurant relative to model-estimated maximal predicted earnings. Horizontal dashed line indicates 100% optimal earnings. Data are presented as the cohort’s (*N* = 31) daily means (±1 SE) across the entire experiment. Color code on the x-axis reflects the stages of training (offer cost ranges denoted from 1 to the number on the top of panel A). Vertical dashed lines (except pink) represent offer block transitions. * on the x-axis indicates immediate significant behavioral change at the block transition. * on the x-axis in (O-P) indicates significantly inefficient decisions (above the 1:1 efficiency ratio line). Otherwise, * indicates gradual significant changes within the 1–30 s block during either the early 2 wk adaptation period or late pink epoch. n.s., not significant; O, offer cost; TL, countdown time left; V_L_, value of time left in countdown at the moment of quitting; V_O_, offer value; VTE, vicarious trial and error; WZ, wait zone threshold.
(TIF)

S2 Fig Decision outcomes as function of offer costs across stages of training. Choice probability to enter versus skip in the offer zone (A-D) or earn versus quit in the wait zone (E-H) relative to all offers (normalized to all session trials) as a function of cost during 1–5 s training (A,E), 1–15 s training (B,F), early 1–30 s training (C,G, first 5 d), and late 1–30 s training (D,H, last 5 d). Vertical dashed lines indicate average threshold. Wait zone thresholds remained relatively stable across 1–15 s and 1–30 s offer blocks. Offer zone thresholds became in register with wait zone thresholds by the end of the 1–30 s training. Horizontal dashed lines indicate choice probability if decisions were made at random. Data are presented as the cohort’s (*N* = 31) means (±1 SE). (I-J) Offer zone outcome (I) and trial-end outcome (J) probabilities as a function of offer cost over the 1–30 s training block (red epoch). All subjects pooled together for visualization purposes. Solid white line represents cohort’s overall average offer zone threshold. Dashed white line represents cohort’s overall average wait zone threshold. Pink line represents onset of food intake and reinforcement rate renormalization after 2 wk of adaptation following the transition to 1–30 s offers (pink epoch spans days 32–70).
(TIF)

S3 Fig Economic characterization of wait zone strategy across stages of training. (A-D) Histogram of wait zone events as a function of time spent waiting and as a function of offer cost. Diagonal unity line (time spent waiting = offer cost) represents earned trials, while the remaining data points represent quit decisions. Horizontal and vertical dashed lines represent average WZs across the 1–5 s block (A), 1–15 s block (B), early 1–30 s block (C, first 5 d), and late 1–30 s block (D, last 5 d). (E-H) Average time spent waiting before quitting as a function of O split by flavor ranking. (I-L) Histogram of quit decisions as a function of V_O_ and V_L_. V_O_ = WZ–O. V_L_ = WZ–TL. O, offer cost; TL, countdown time left; V_L_, value of time left in countdown at the moment of quitting; V_O_, offer value; WZ, wait zone threshold
(TIF)

S4 Fig Development of deliberative decisions as a function of V_O_ across training. (A-B) Offer zone reaction time (A) and VTE behavior (B) as a function of V_O_ (V_O_ = WZ–O) over days of learning in the 1–30 s offer block (red epoch). Blue line represents 0 value trials (where offer = WZ). Pink line represents onset of food intake and reinforcement rate renormalization after 2 wk of adaptation following the transition to 1–30 s offers (pink epoch spans days 32–70). Graphical projection against the back wall displays data presented as the cohort’s (*N* = 31) daily means (±1 SE) during the last 5 d of training (days 65–70). Z-axis is redundant with color scale for visualization purposes. (C-D) Days 65–70 offer zone time (C) and VTE (D) as a function of V_O_ split by flavor rank. Vertical dashed black lines represent 0 value trials. O, offer cost; V_O_, offer value; VTE, vicarious trial and error; WZ, wait zone threshold
(TIF)

S5 Fig Signal detection theory approach to characterize the development of deliberative value-based discriminability and biases. (A) Offer zone decision distributions as a function of V_O_ (V_O_ = WZ–O) split by enter versus skip decisions. As a function of a sliding R.O.C. criterion, R.O.C. curves (B) can be generated by plotting calculated hit rate and false-alarm-rate pairs at each liberal-to-conservative sliding R.O.C. criterion. Relative to each sliding R.O.C. criterion, hits, misses, false alarms, and correct rejections are characterized by enter versus skip outcomes for offers whose values lie either to the left or right of the R.O.C. criterion. Economic violations (“X’s”) represent either “misses” in incorrectly detected criterion-relative negatively valued offers (thus, entering) or “F.A.’s” in incorrectly detected criterion-relative positively valued offers as criterion-relative negatively valued offers (thus, skipping). Hits represent correctly detected criterion-relative positively valued offers (thus, entering), and C.R.’s represent correctly detected criterion-relative negatively valued offers (thus, skipping). Hit rate = hits / total enters. F.A. rate = F.A.’s / total skips. (B) Offer zone R.O.C. curves changes from being linear (day 18, chance-decision-maker) to bowed-shaped (day 70, good-value-based-signal-detector) quantified by an increase in A.U.C. (solid red line indicates chance unity line with 0.5 A.U.C.). (C) Offer zone R.O.C. A.U.C. plotted across days of training in the 1–30 s offer block (red epoch) split by flavor ranking. Vertical pink line represents onset of food intake and reinforcement rate renormalization after 2 wk of adaptation following the transition to 1–30 s offers (pink epoch spans days 32–70). (D) Offer zone R.O.C. curve skew describes a value bias (tail) of either enter or skip distributions. This is evident in (A) by the (-) left tail of the enter distribution and reflected in (B) by an asymmetry of R.O.C. curve bowedness. Dashed red diagonal line in (B) aids in visualization of R.O.C. curve asymmetry. Gaussian fit peak deviation from this line is quantified in (D). (E-H) reflect same analyses in wait zone decisions. Data (C-D) and (G-H) presented as the cohort’s (*N* = 31) daily means (±1 SE). * indicate significant change over 1–30 s offer block. A.U.C., area under the curve; C.R., correct rejection; F.A., false alarm; n.s., not significant; O, offer cost; R.O.C., receiver operating characteristic; V_O_, offer value; WZ, wait zone threshold
(TIF)

S6 Fig Controlling for flavor preferences in regret-like sequence effects. To control for potential differences in restaurant sequences due to the identity of flavor preferences in Restaurant 2 following quits versus skips in Restaurant 1, we sorted scenarios such that the Restaurant 2 was always either the least or most preferred flavor. (A) Probability of entering an offer in Restaurant 2 after skipping versus quitting in Restaurant 1. Augmented by quits (increased) versus skips in only least preferred restaurants. (B) Offer zone reaction time in Restaurant 2 after skipping versus quitting in Restaurant 1. Augmented by quits (decreased) versus skips in both least and most preferred restaurants. (C) Time spent consuming an earned pellet and lingering at the reward site in Restaurant 2 after skipping versus quitting in Restaurant 1. Augmented by quits (decreased) versus skips in only most preferred restaurants. Data averaged across the 1–30 s offer block. * indicate significant difference between skip versus quit conditions.
(TIF)

S7 Fig Visualization of offer-length distributions between skip and quit events. (A) Histogram of offer length distributions comparing trials that ended as skips versus quits from data pooled across animals from days 60–70. (B) Samples were randomly selected from the skip distribution to match the number of samples from the quit distribution. Skip resampling was bootstrapped 100 times and replotted in (B). These data indicate both trial types derive from the same offer length distributions.
(TIF)

S8 Fig Controlling for the effects of VTE on reinforcement rate. Reinforcement rate (inter-earn-interval) is plotted across days, comparing observed date (black) versus 4 different computer models that simulated what the expected reinforcement rate would be if high-VTE trials were adjusted. High- versus low-VTE trials were determined by a median split of VTE values taken across the entire experiment. The removal simulation (red) simply removed high-VTE trials before reinforcement rates were calculated. The 3 replacement simulations (cyan, blue, purple) resampled trial outcomes from low-VTE trials and differed based on how offer length was resampled when earned trials were simulated (offer length retained from the high-VTE trial, offer length randomly selected from the distribution for low-VTE trials, or offer length randomly selected from the uniform range of offers for that block, respectively). These simulations indicate no contributions to reinforcement rate due to high-VTE trials during the early 1–30 s epoch, despite having an effect late into 1–30 s training. Data presented as the cohort’s (*N* = 31) daily means (±1 SE). Color code on the x-axis reflects the stages of training (offer cost ranges denoted from 1 to the number on the top of the plot). * indicates significant difference compared against observed data. n.s., not significant; VTE, vicarious trial and error.
(TIF)

S1 Text Supplemental analyses and discussion. Additional analyses and discussion are available in the supplemental text, including analyses and discussion on (1) early conditioned place behaviors; (2) the development of default responses in the reward-rich components of training; (3) how demand elasticity changed across the longitudinal design; (4) evidence that the mice behaved suboptimally on this task; (5) postregret compensatory valuations; and (6) the relationship between the reluctance to skip, the development of deliberative strategies as the environment became reward-scarce, and VTE behaviors. VTE, vicarious trial and error.
(DOCX)

S1 Video Example behavior on Restaurant Row. In this excerpt, a top-down view of the maze is presented. The mouse’s position is tracked automatically at center-of-mass (orange dot). The 4 corners of the maze represent the 4 restaurants, each fixed in location with unique visual patterns on the walls (chocolate vertical stripes, banana checker, grape triangle, plain horizontal stripes). Orange lines on the maze represent computer-overlaid boundaries separating offer zones, wait zones, and between-restaurant corridors. Tones sounded upon offer zone entry repeated with a fixed interval but did not descend in pitch (“count-down”) until entry into the wait zone (also at the same fixed interval). Note the VTE behaviors shown in the offer zone and the quit event occurring in the last example in the grape restaurant. Video is from day 70 of a well-trained mouse. VTE, vicarious trial and error.
(MP4)

## Abbreviations

IACUC: Institutional Animal Care and Use Committee
TL: countdown time left
V_L_: value of time left in countdown at the moment of quitting
V_O_: offer value
VTE: vicarious trial and error

## References

[1] Zeelenberg M, Pieters R. A theory of regret regulation 1.0. J Consumer Psych. 2007;17:3–18.

[2] Coricelli G, Critchley HD, Joffily M, O'Doherty JP, Sirigu A, Dolan RJ. Regret and its avoidance: a neuroimaging study of choice behavior. Nat Neurosci. 2005;8(9):1255–1262. doi: 10.1038/nn1514 16116457 10.1038/nn1514

[3] Abe H, Lee D. Distributed coding of actual and hypothetical outcomes in the orbital and dorsolateral prefrontal cortex. Neuron. 2011;70:731–741. doi: 10.1016/j.neuron.2011.03.026 21609828 10.1016/j.neuron.2011.03.026 PMC3104017

[4] Steiner A, Redish AD. Behavioral and neurophysiological correlates of regret in rat decision-making on a neuroeconomic task. Nature Neuroscience. 2014;17:995–1002. doi: 10.1038/nn.3740 24908102 10.1038/nn.3740 PMC4113023

[5] Epstude K, Roese NJ. The functional theory of counterfactual thinking. Pers Soc Psychol Rev. 2008;12:168–92. doi: 10.1177/1088868308316091 18453477 10.1177/1088868308316091 PMC2408534

[6] Byrne R. Mental models and counterfactual thoughts about what might have been. Trends Cogn Sci. 2002;6:426–431. 12413576 10.1016/s1364-6613(02)01974-5

[7] Coricelli G, Rustichini A. Counterfactual thinking and emotions: regret and envy learning. Phil Trans Royal Soc B. 2010;365:241–247. 10.1098/rstb.2009.0159 PMC2827450 20026462

[8] Frydman C, Camerer C. Neural evidence of regret and its implications for investor behavior. The Review of Financial Studies. 2016;29:3108–3139.

[9] Dickhaut J, Rustichini A. A neuroeconomic theory of the decision process. PNAS. 2009;106(52):22145–22150. doi: 10.1073/pnas.0912500106 20080787 10.1073/pnas.0912500106 PMC2799727

[10] Loewenstein G, Rick S, Cohen J. Neuroeconomics. Annual review of psychology. 2008;59:647–72. doi: 10.1146/annurev.psych.59.103006.093710 17883335 10.1146/annurev.psych.59.103006.093710

[11] Kalenscher T, van Wingerden M. Why we should use animals to study economic decision making—a perspective. Front Neurosci. 2011;5:82 doi: 10.3389/fnins.2011.00082 21731558 10.3389/fnins.2011.00082 PMC3118901

[12] Rangel A, Camerer C, Montague R. A framework for studying the neurobiology of value-based decision making. Nature Reviews Neuroscience. 2008;9:545–556. doi: 10.1038/nrn2357 18545266 10.1038/nrn2357 PMC4332708

[13] Steiner A, Redish AD. The road not taken: neural correlates of decision making in orbitofrontal cortex. Frontiers in Neuroscience. 2012;6:131 doi: 10.3389/fnins.2012.00131 22973189 10.3389/fnins.2012.00131 PMC3438732

[14] Camille N, Coricelli G, Sallet J, Pradat-Diehl P, Duhamel JR, Sirigu A. The involvement of the orbitofrontal cortex in the experience of regret. Science. 2004;304:1167–1170. doi: 10.1126/science.1094550 15155951 10.1126/science.1094550

[15] Sommer T, Peters J, Gläscher J, Büchel C. Structure–function relationships in the processing of regret in the orbitofrontal cortex. Brain Struct Funct. 2009;213:535–551. doi: 10.1007/s00429-009-0222-8 19760243 10.1007/s00429-009-0222-8

[16] Redish AD. Vicarious trial and error. Nat. Rev. Neurosci. 2016;17:147–59. doi: 10.1038/nrn.2015.30 26891625 10.1038/nrn.2015.30 PMC5029271

[17] Tolman EC. Prediction of vicarious trial and error by means of the schematic sowbug. Psychological Review. 1939;46:318–336.

[18] Muenzinger KF. On the origin and early use of the term vicarious trial and error (VTE). Psychological Bulletin. 1956;53:493–4. 13370695 10.1037/h0044135

[19] Johnson A, Redish AD. Neural ensembles in CA3 transiently encode paths forward of the animal at a decision point. Journal of Neuroscience. 2007;27(45):12176–12189. doi: 10.1523/JNEUROSCI.3761-07.2007 17989284 10.1523/JNEUROSCI.3761-07.2007 PMC6673267

[20] Van der Meer MAA, Redish AD. Theta phase precession in rat ventral striatum links place and reward information. Journal of Neuroscience. 2011;31:2843–54. doi: 10.1523/JNEUROSCI.4869-10.2011 21414906 10.1523/JNEUROSCI.4869-10.2011 PMC3758553

[21] Van der Meer MAA, Redish AD. Covert expectation-of-reward in rat ventral striatum at decision points. Frontiers in Integrative Neuroscience. 2009;3(1):1–15. 19225578 10.3389/neuro.07.001.2009 PMC2644619

[22] Van der Meer MAA, Johnson A, Schmitzer-Torbert N, RedishAD. Triple dissociation of information processing in dorsal striatum, ventral striatum, and hippocampus on a learned spatial decision task. Neuron. 2010;67:25–32. doi: 10.1016/j.neuron.2010.06.023 20624589 10.1016/j.neuron.2010.06.023 PMC4020415

[23] Stott J, Redish AD. A functional difference in information processing between orbitofrontal cortex and ventral striatum during decision-making behaviour. Phil Trans Royal Soc B. 2014;369:20130472. 10.1098/rstb.2013.0472 PMC4186226 25267815

[24] Papale A, Stott J, Powell N, Regier P, Redish AD. Interactions between deliberation and delay-discounting in rats. CABN. 2012;12:513–26. doi: 10.3758/s13415-012-0097-7 22588853 10.3758/s13415-012-0097-7 PMC3774285

[25] Papale AE, Zielinski MC, Frank LM, Jadhav SP, Redish AD. Interplay between hippocampal sharp-wave-ripple events and vicarious trial and error behaviors in decision making. Neuron. 2016;92:975–982. doi: 10.1016/j.neuron.2016.10.028 27866796 10.1016/j.neuron.2016.10.028 PMC5145752

[26] Loomes G, Surgden R. Regret theory: An alternative theory of rational choice under uncertainty. Economic Journal. 1982;92:805–824.

[27] Patrick VM, Lancellotti MP, Demello G. Coping with non-purchase: Managing the stress of inaction regret. Journal of Consumer Psychology. 2009;19:463–472.

[28] Bell DE. Regret in decision making under uncertainty. Operational Research. 1982;30:961–981.

[29] Knutson B, Greer S. Anticipatory affect: neural correlates and consequences for choice. Phil Trans Royal Soc B. 2008;363:3771–3786. 10.1098/rstb.2008.0155 PMC2607363 18829428

[30] Blanchard T, Hayden B. Neurons in dorsal anterior cingulate cortex signal postdecisional variables in a foraging task. J Neurosci. 2014;34:646–655. doi: 10.1523/JNEUROSCI.3151-13.2014 24403162 10.1523/JNEUROSCI.3151-13.2014 PMC3870941

[31] Marchiori D, Warglien M. Predicting human interactive learning by regret-driven neural networks. Science. 2008;319:1111–1113. doi: 10.1126/science.1151185 18292345 10.1126/science.1151185

[32] Kolling N, Akam T. (Reinforcement?) Learning to forage optimally. Curr Opin Neurobiol. 2017;46:162–169. doi: 10.1016/j.conb.2017.08.008 28918312 10.1016/j.conb.2017.08.008

[33] Dayan P, Niv Y. Reinforcement learning: The good, the bad and the ugly. Curr Opin Neurobiol. 2008;18:185–196. doi: 10.1016/j.conb.2008.08.003 18708140 10.1016/j.conb.2008.08.003

[34] Ainslie G. Specious reward: A behavioral theory of impulsiveness and impulse control. Psychological Bulletin; 1975; 82(4):463–496. 1099599 10.1037/h0076860

[35] Stephens D, Krebs J. Foraging Theory. Princeton: Princeton Univ Press; 1987.

[36] Sutton RS, Barto AG. Reinforcement learning: An introduction. Cambridge: MIT Press; 1998.

[37] Holroyd CB, Coles MGH. The neural basis of human error processing: reinforcement learning, dopamine, and the error-related negativity. Psychol Rev. 2002;109:679–709. doi: 10.1037/0033-295X.109.4.679 12374324 10.1037/0033-295X.109.4.679

[38] Suri RE, Schultz W. A neural network model with dopamine-like reinforcement signal that learns a spatial delayed response task. Neuroscience. 1999;91(3):871–90. 10391468 10.1016/s0306-4522(98)00697-6

[39] Ko D, Wanat MJ. Phasic dopamine transmission reflects initiation vigor and exerted effort in an action- and region-specific manner. J. Neurosci. 2016;36:2202–11. doi: 10.1523/JNEUROSCI.1279-15.2016 26888930 10.1523/JNEUROSCI.1279-15.2016 PMC4756155

[40] Schultz W. Reward prediction error. Curr. Biol. 2017;27:369–371. 10.1016/j.cub.2017.02.064 28535383

[41] Schelp SA, Pultorak KJ, Rakowski DR, Gomez DM, Krzystyniak G, Das R, et al A transient dopamine signal encodes subjective value and causally influences demand in an economic context. PNAS. 2017;114(52):E11303–E11312. doi: 10.1073/pnas.1706969114 29109253 10.1073/pnas.1706969114 PMC5748169

[42] Shizgal P. Neural basis of utility estimation. Curr. Opin. Neurobiol. 1997;7:198–208. 9142755 10.1016/s0959-4388(97)80008-6

[43] Ahn H, Picard RW. Affective-cognitive learning and decision making: A motivational reward framework for affective agents. International Conference on Affective Computing and Intelligent Interaction. 2005;866–873.

[44] Kim H, Shimojo S, O’Doherty JP. Is avoiding an aversive outcome rewarding? Neural substrates of avoidance learning in the human brain. PLoS Biol. 2006;4(8):1453–1461. 10.1371/journal.pbio.0040233 PMC1484497 16802856

[45] Kim JJ, Jung MW. Neural circuits and mechanisms involved in Pavlovian fear conditioning: A critical review. Neuroscience Biobehav Rev. 2006;30(2):188–202. 10.1016/j.neubiorev.2005.06.005 PMC4342048 16120461

[46] Krypotos AM, Effting M, Kindt M, Beckers T. Avoidance learning: a review of theoretical models and recent developments. Front Behav Neurosci. 2015;9(189):1–16. 26257618 10.3389/fnbeh.2015.00189 PMC4508580

[47] Resulaj A, Kiani R, Wolpert D, Shadlen M. Changes of mind in decision-making. Nature. 2009;461:263–6. doi: 10.1038/nature08275 19693010 10.1038/nature08275 PMC2875179

[48] Van den Berg R, Anandalingam K, Zylberberg A, Kiani R, Shadlen MN, Wolpert DM. A common mechanism underlies changes of mind about decisions and confidence. eLife. 2016;5:e12192 doi: 10.7554/eLife.12192 26829590 10.7554/eLife.12192 PMC4798971

[49] Churchland A, Kiani R, Shadlen M. Decision-making with multiple alternatives. Nature Neuroscience. 2008;11:693–702. doi: 10.1038/nn.2123 18488024 10.1038/nn.2123 PMC2453226

[50] Wilson TD, Gilbert DT. Affective forecasting: Knowing what to want. Current Directions in Psychological Science. 2005;14(3):131–134.

[51] Gilbert DT, Ebert JE. Decisions and revisions: The affective forecasting of changeable outcomes. Journal of Pers Soc Psychol. 2002;82(4):503–514. 11999920

[52] Kermer DA, Driver-Linn E, Wilson TD, Gilbert DT. Loss aversion is an affective forecasting error. Psychological Sci. 2006;17(8):649–53. 10.1111/j.1467-9280.2006.01760.x 16913944

[53] Roese NJ, Summerville A. What we regret most … and why. Pers Soc Psychol Bull. 2005;31(9):1273–1285. doi: 10.1177/0146167205274693 16055646 10.1177/0146167205274693 PMC2394712

[54] Arkes H, Blumer C. The psychology of sunk cost. Organ Behav Hum 12 1985;35:124–140.

[55] Sweis BM, Abram SV, Schmidt BJ, Breton YA, MacDonald AW, Thomas MJ, Redish AD. Sunk cost effects appear similar in parallel neuroeconomic foraging tasks in mice, rats, and humans. Society for Neuroeconomics. 2017;2-1-26.

