# Supplementary figures and images for: Mice learn to avoid regret
Source: PLoS Biol. 2018 Jun 21;16(6):e2005853. doi: 10.1371/journal.pbio.2005853 (PMC6013153; doi:10.1371/journal.pbio.2005853)

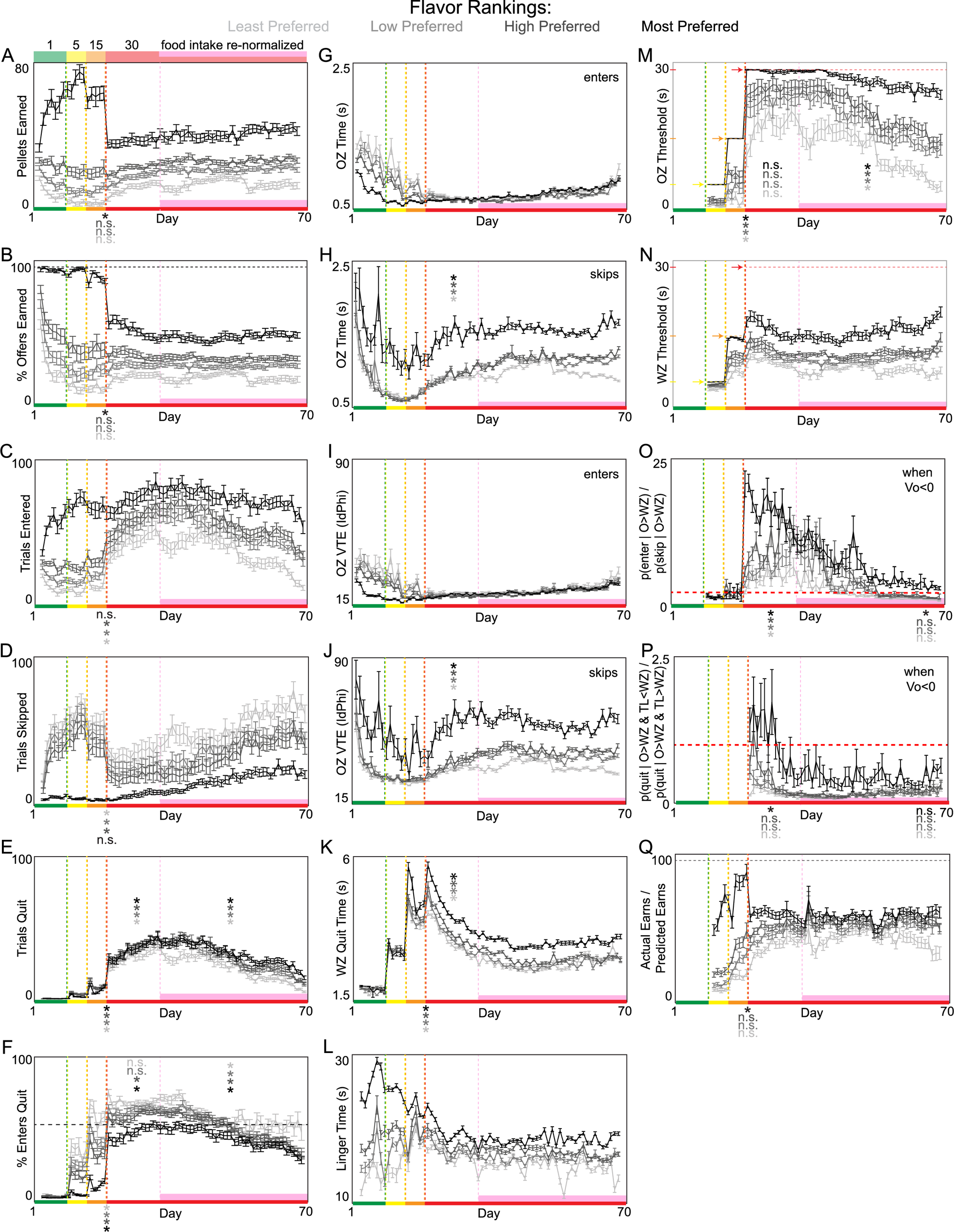

Supplement: S1 Fig — Flavors were ranked from least preferred to most preferred based on total pellet earnings in each restaurant at the end of each session. (A) Pellets earned in each restaurant show early development of flavor preferences that persist throughout the entire experiment. (B) Percentage of offers entered. Horizontal dashed line indicates 100%. (C-E) Total number of trials entered (C), skipped (D), and quit (E). (F) Percentage of entered offers quit. Horizontal dashed line indicates 50%. (G-J) Offer zone behaviors for enter (G, time; I, VTE) and skip (H, time; J, VTE) decisions. (K) Time spent in the wait zone during tone countdown before quitting. (L) Time spent in the wait zone consuming an earned food pellet and lingering near the reward site before advancing to the next trial. (M-N) Offer zone (M) and wait zone (N) thresholds. Horizontal dashed lines represent the maximum possible threshold in each block. (O) Offer zone inefficiency ratio. VO = WZ–O. Probability of entering negatively valued offers relative to the probability of skipping negatively valued offers. Horizontal dashed line indicates equivalent 1:1 ratio of entering versus skipping negatively valued offers. (P) Wait zone inefficiency ratio. VL = WZ–TL. Probability of quitting negatively valued offers when VL was positive relative to when VL was still negative. Horizontal dashed line indicates equivalent 1:1 ratio of quitting inefficiently versus efficiently. (Q) Reward-earning optimality. Proportion of pellets mice actually earned in each restaurant relative to model-estimated maximal predicted earnings. Horizontal dashed line indicates 100% optimal earnings. Data are presented as the cohort’s (N = 31) daily means (±1 SE) across the entire experiment. Color code on the x-axis reflects the stages of training (offer cost ranges denoted from 1 to the number on the top of panel A). Vertical dashed lines (except pink) represent offer block transitions. * on the x-axis indicates immediate significant behavioral c [file pbio.2005853.s001.tif]

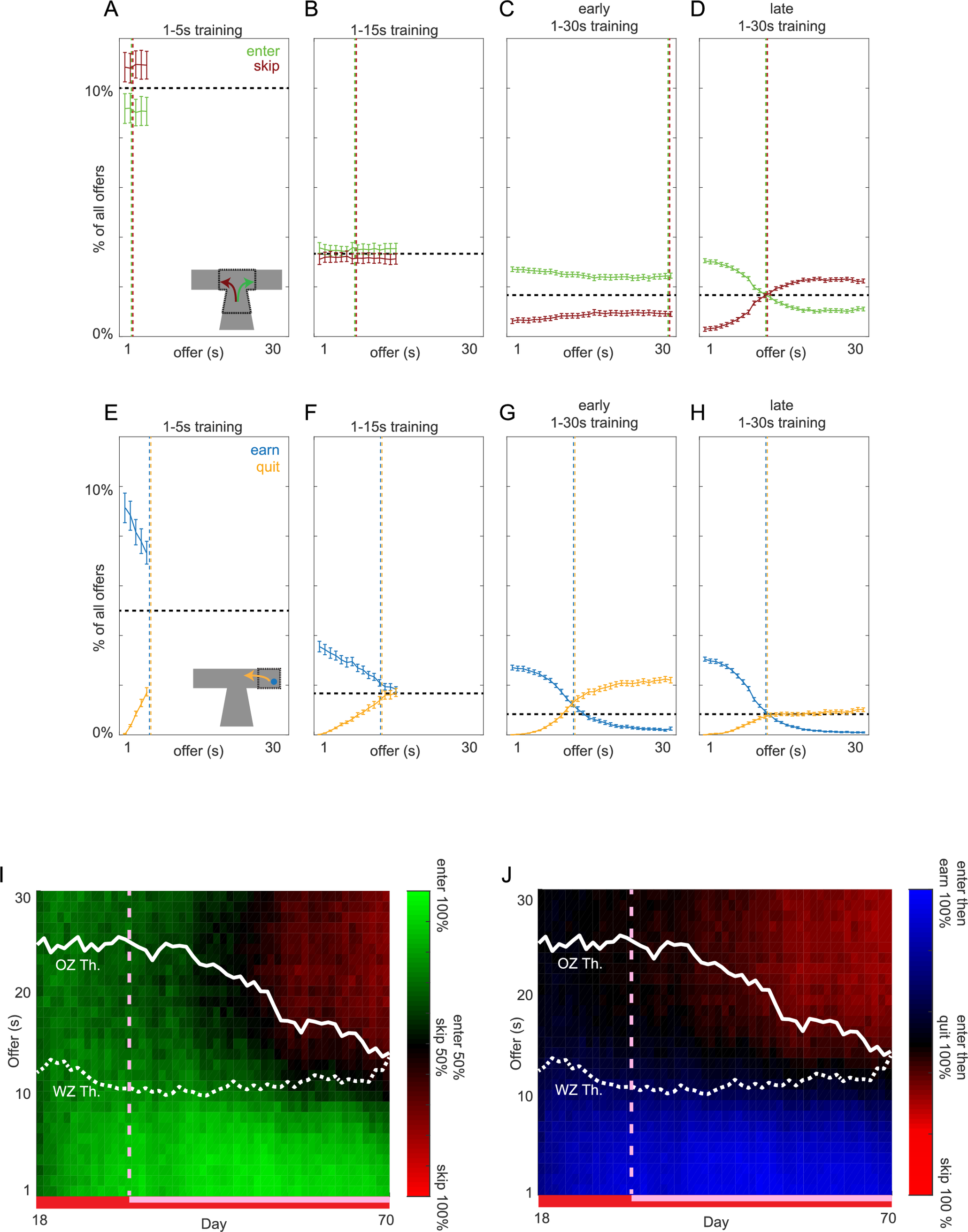

Supplement: S2 Fig — Choice probability to enter versus skip in the offer zone (A-D) or earn versus quit in the wait zone (E-H) relative to all offers (normalized to all session trials) as a function of cost during 1–5 s training (A,E), 1–15 s training (B,F), early 1–30 s training (C,G, first 5 d), and late 1–30 s training (D,H, last 5 d). Vertical dashed lines indicate average threshold. Wait zone thresholds remained relatively stable across 1–15 s and 1–30 s offer blocks. Offer zone thresholds became in register with wait zone thresholds by the end of the 1–30 s training. Horizontal dashed lines indicate choice probability if decisions were made at random. Data are presented as the cohort’s (N = 31) means (±1 SE). (I-J) Offer zone outcome (I) and trial-end outcome (J) probabilities as a function of offer cost over the 1–30 s training block (red epoch). All subjects pooled together for visualization purposes. Solid white line represents cohort’s overall average offer zone threshold. Dashed white line represents cohort’s overall average wait zone threshold. Pink line represents onset of food intake and reinforcement rate renormalization after 2 wk of adaptation following the transition to 1–30 s offers (pink epoch spans days 32–70). (TIF) [file pbio.2005853.s002.tif]

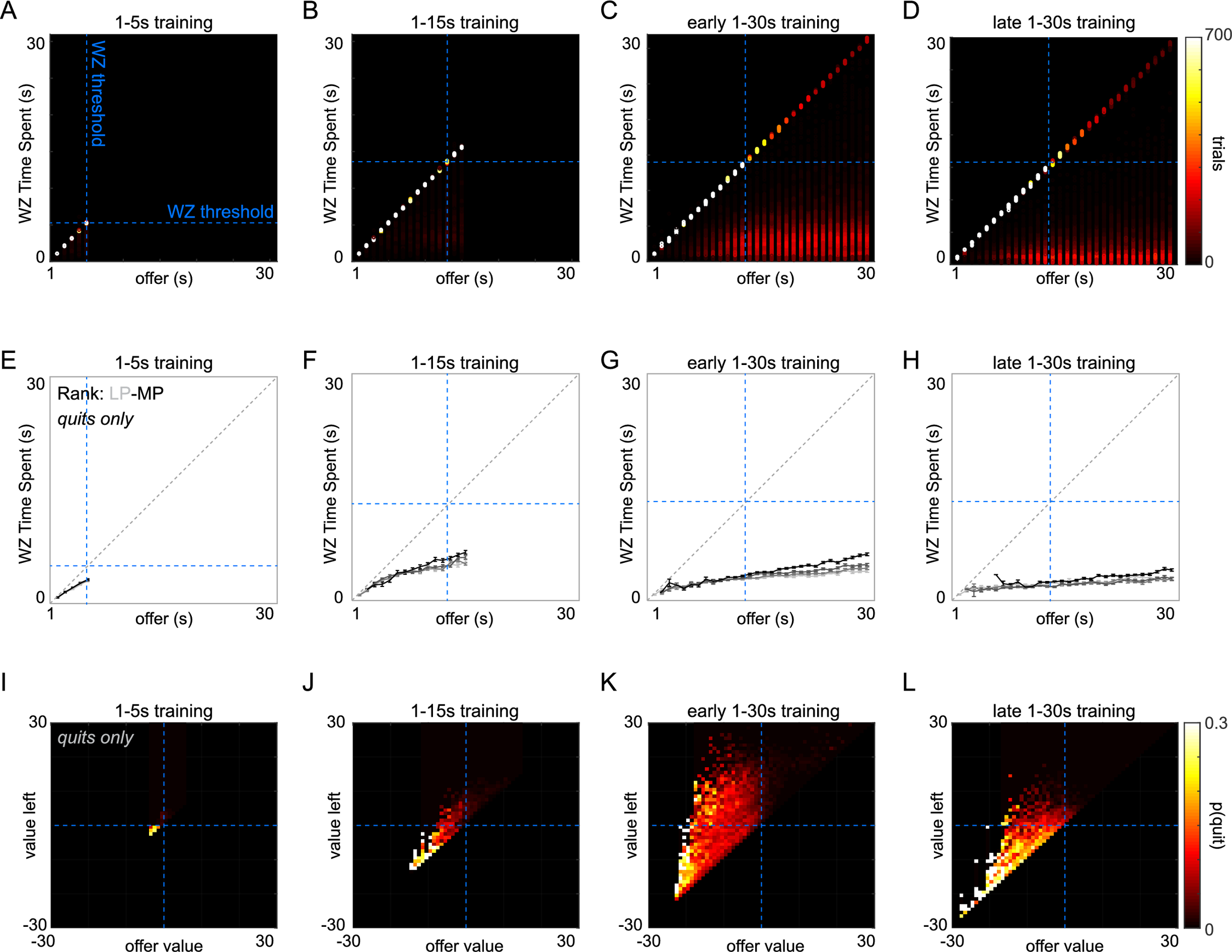

Supplement: S3 Fig — (A-D) Histogram of wait zone events as a function of time spent waiting and as a function of offer cost. Diagonal unity line (time spent waiting = offer cost) represents earned trials, while the remaining data points represent quit decisions. Horizontal and vertical dashed lines represent average WZs across the 1–5 s block (A), 1–15 s block (B), early 1–30 s block (C, first 5 d), and late 1–30 s block (D, last 5 d). (E-H) Average time spent waiting before quitting as a function of O split by flavor ranking. (I-L) Histogram of quit decisions as a function of VO and VL. VO = WZ–O. VL = WZ–TL. O, offer cost; TL, countdown time left; VL, value of time left in countdown at the moment of quitting; VO, offer value; WZ, wait zone threshold (TIF) [file pbio.2005853.s003.tif]

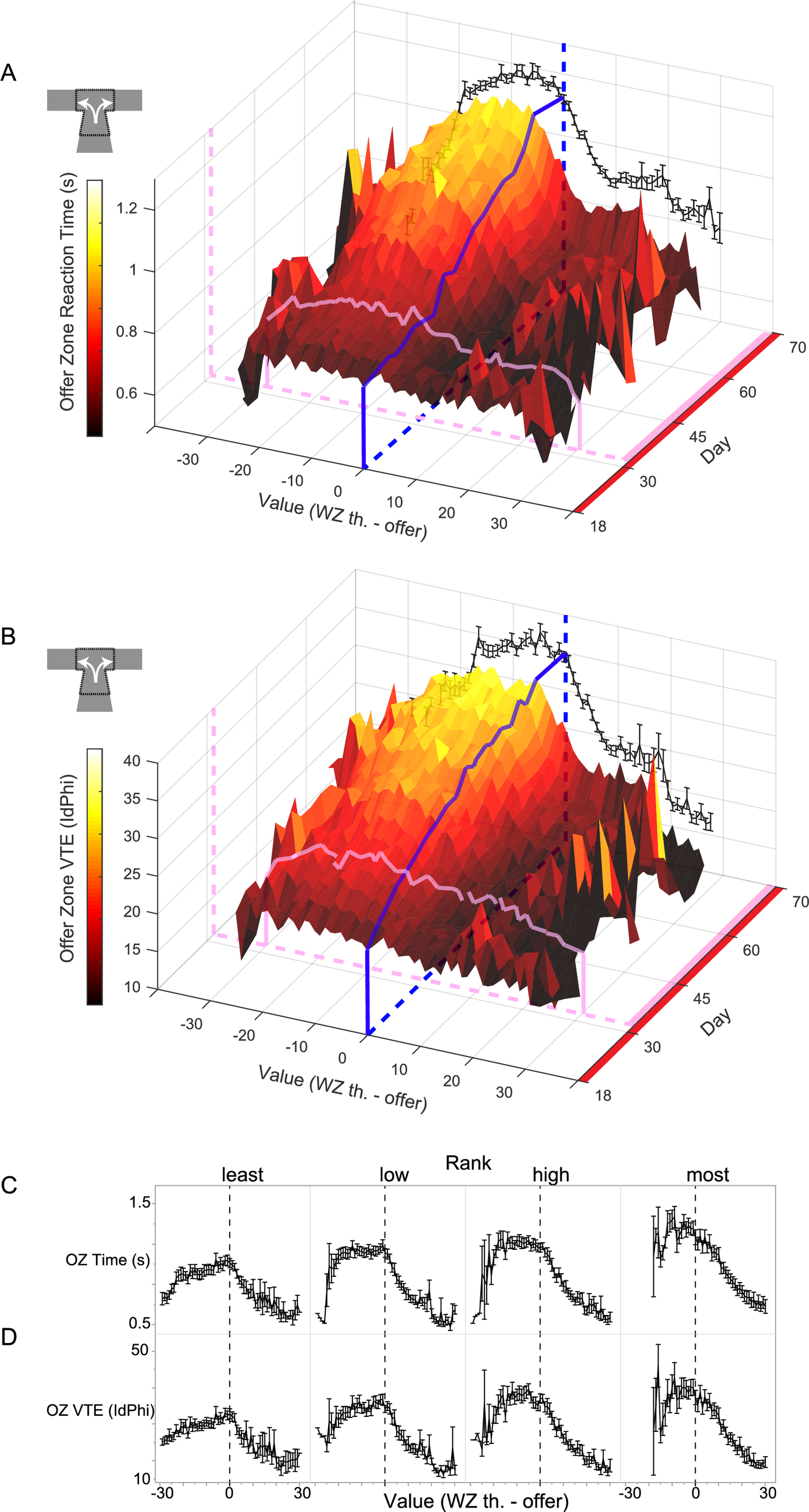

Supplement: S4 Fig — (A-B) Offer zone reaction time (A) and VTE behavior (B) as a function of VO (VO = WZ–O) over days of learning in the 1–30 s offer block (red epoch). Blue line represents 0 value trials (where offer = WZ). Pink line represents onset of food intake and reinforcement rate renormalization after 2 wk of adaptation following the transition to 1–30 s offers (pink epoch spans days 32–70). Graphical projection against the back wall displays data presented as the cohort’s (N = 31) daily means (±1 SE) during the last 5 d of training (days 65–70). Z-axis is redundant with color scale for visualization purposes. (C-D) Days 65–70 offer zone time (C) and VTE (D) as a function of VO split by flavor rank. Vertical dashed black lines represent 0 value trials. O, offer cost; VO, offer value; VTE, vicarious trial and error; WZ, wait zone threshold (TIF) [file pbio.2005853.s004.tif]

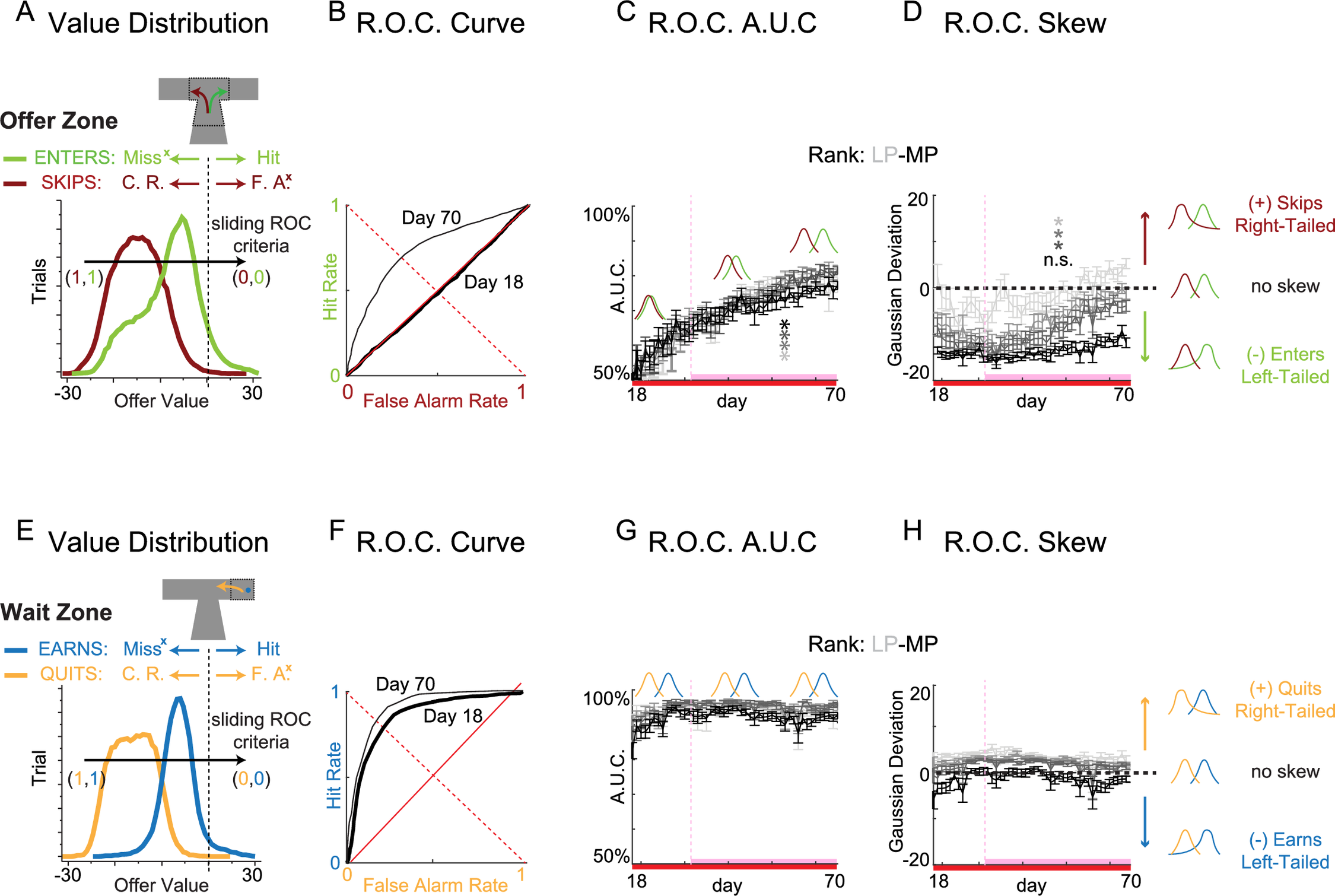

Supplement: S5 Fig — (A) Offer zone decision distributions as a function of VO (VO = WZ–O) split by enter versus skip decisions. As a function of a sliding R.O.C. criterion, R.O.C. curves (B) can be generated by plotting calculated hit rate and false-alarm-rate pairs at each liberal-to-conservative sliding R.O.C. criterion. Relative to each sliding R.O.C. criterion, hits, misses, false alarms, and correct rejections are characterized by enter versus skip outcomes for offers whose values lie either to the left or right of the R.O.C. criterion. Economic violations (“X’s”) represent either “misses” in incorrectly detected criterion-relative negatively valued offers (thus, entering) or “F.A.’s” in incorrectly detected criterion-relative positively valued offers as criterion-relative negatively valued offers (thus, skipping). Hits represent correctly detected criterion-relative positively valued offers (thus, entering), and C.R.’s represent correctly detected criterion-relative negatively valued offers (thus, skipping). Hit rate = hits / total enters. F.A. rate = F.A.’s / total skips. (B) Offer zone R.O.C. curves changes from being linear (day 18, chance-decision-maker) to bowed-shaped (day 70, good-value-based-signal-detector) quantified by an increase in A.U.C. (solid red line indicates chance unity line with 0.5 A.U.C.). (C) Offer zone R.O.C. A.U.C. plotted across days of training in the 1–30 s offer block (red epoch) split by flavor ranking. Vertical pink line represents onset of food intake and reinforcement rate renormalization after 2 wk of adaptation following the transition to 1–30 s offers (pink epoch spans days 32–70). (D) Offer zone R.O.C. curve skew describes a value bias (tail) of either enter or skip distributions. This is evident in (A) by the (-) left tail of the enter distribution and reflected in (B) by an asymmetry of R.O.C. curve bowedness. Dashed red diagonal line in (B) aids in visualization of R.O.C. curve asymmetry. Gaussian fit peak deviation from this line is quant [file pbio.2005853.s005.tif]

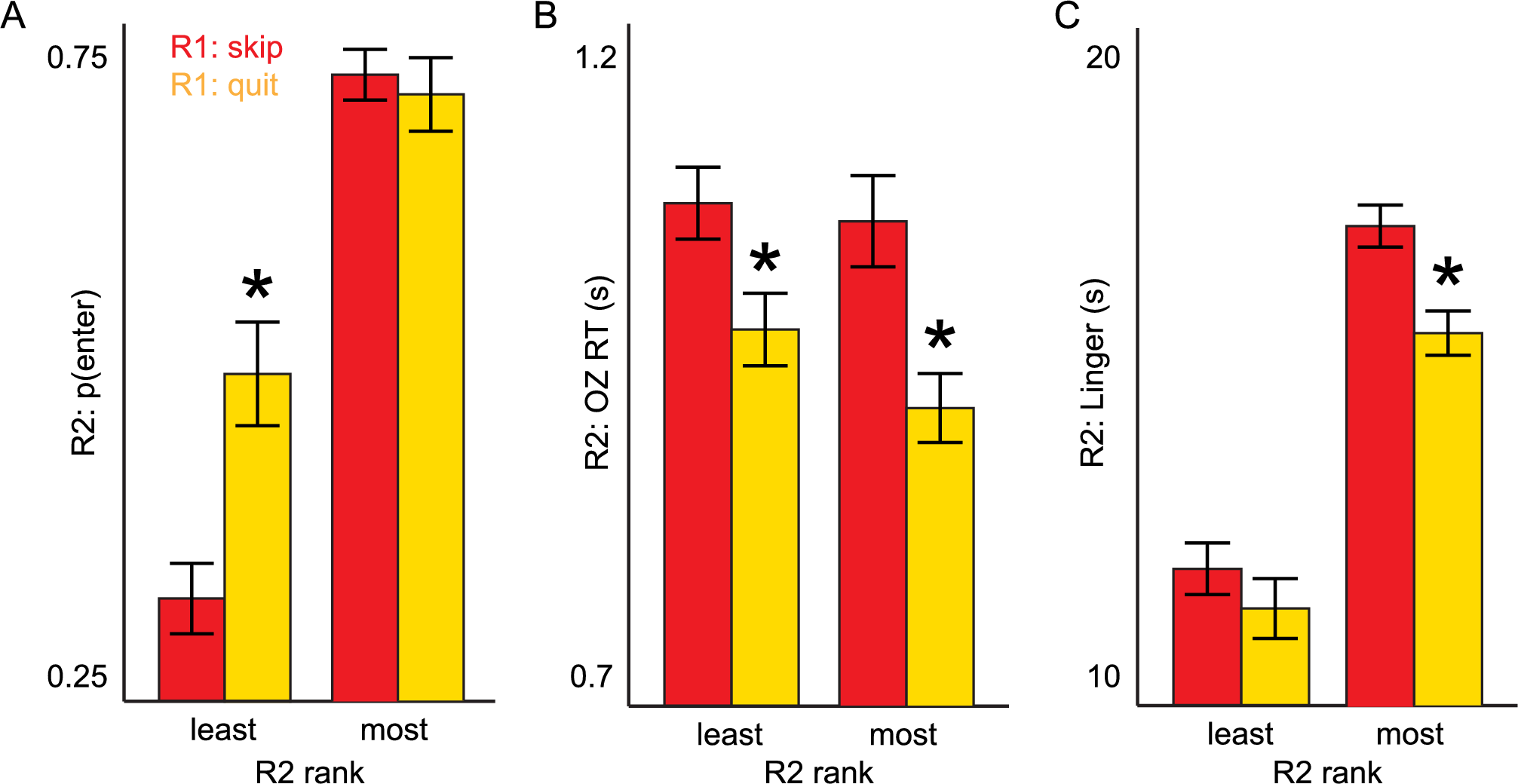

Supplement: S6 Fig — To control for potential differences in restaurant sequences due to the identity of flavor preferences in Restaurant 2 following quits versus skips in Restaurant 1, we sorted scenarios such that the Restaurant 2 was always either the least or most preferred flavor. (A) Probability of entering an offer in Restaurant 2 after skipping versus quitting in Restaurant 1. Augmented by quits (increased) versus skips in only least preferred restaurants. (B) Offer zone reaction time in Restaurant 2 after skipping versus quitting in Restaurant 1. Augmented by quits (decreased) versus skips in both least and most preferred restaurants. (C) Time spent consuming an earned pellet and lingering at the reward site in Restaurant 2 after skipping versus quitting in Restaurant 1. Augmented by quits (decreased) versus skips in only most preferred restaurants. Data averaged across the 1–30 s offer block. * indicate significant difference between skip versus quit conditions. (TIF) [file pbio.2005853.s006.tif]

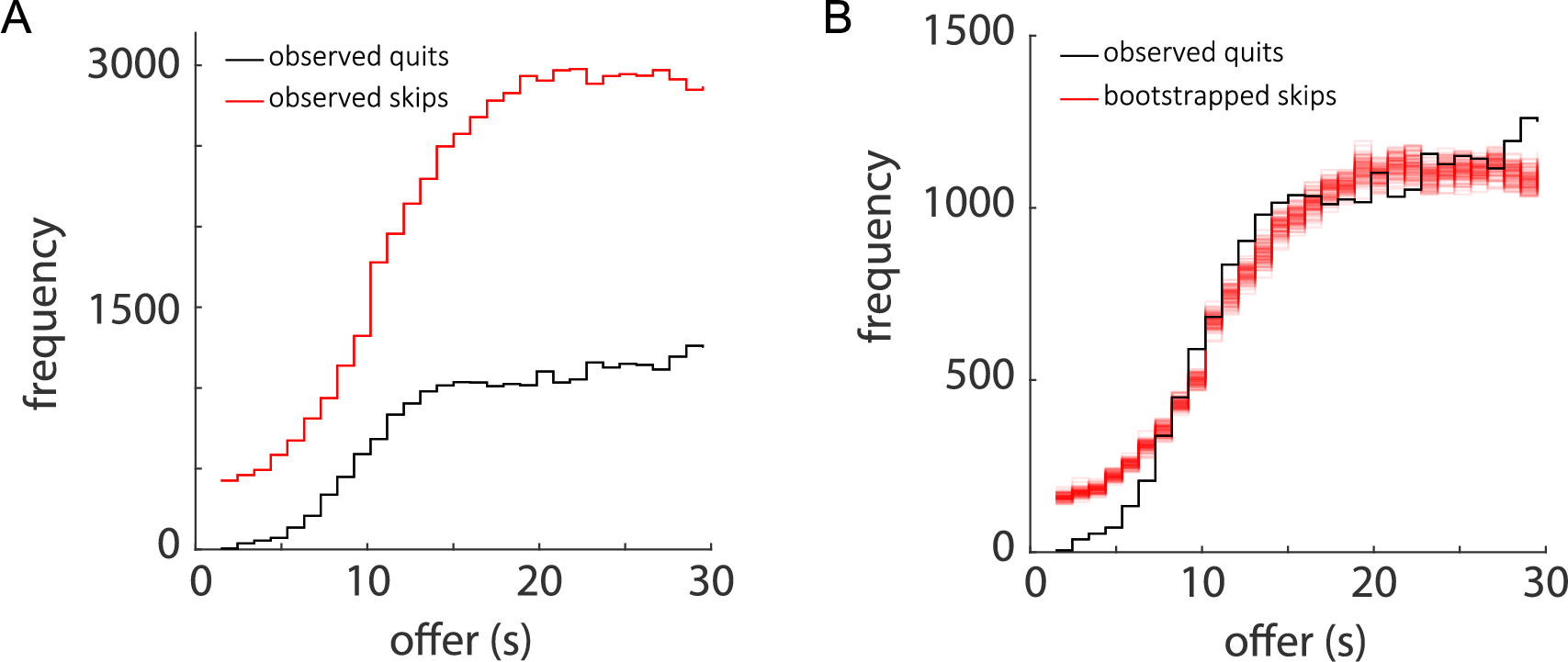

Supplement: S7 Fig — (A) Histogram of offer length distributions comparing trials that ended as skips versus quits from data pooled across animals from days 60–70. (B) Samples were randomly selected from the skip distribution to match the number of samples from the quit distribution. Skip resampling was bootstrapped 100 times and replotted in (B). These data indicate both trial types derive from the same offer length distributions. (TIF) [file pbio.2005853.s007.tif]

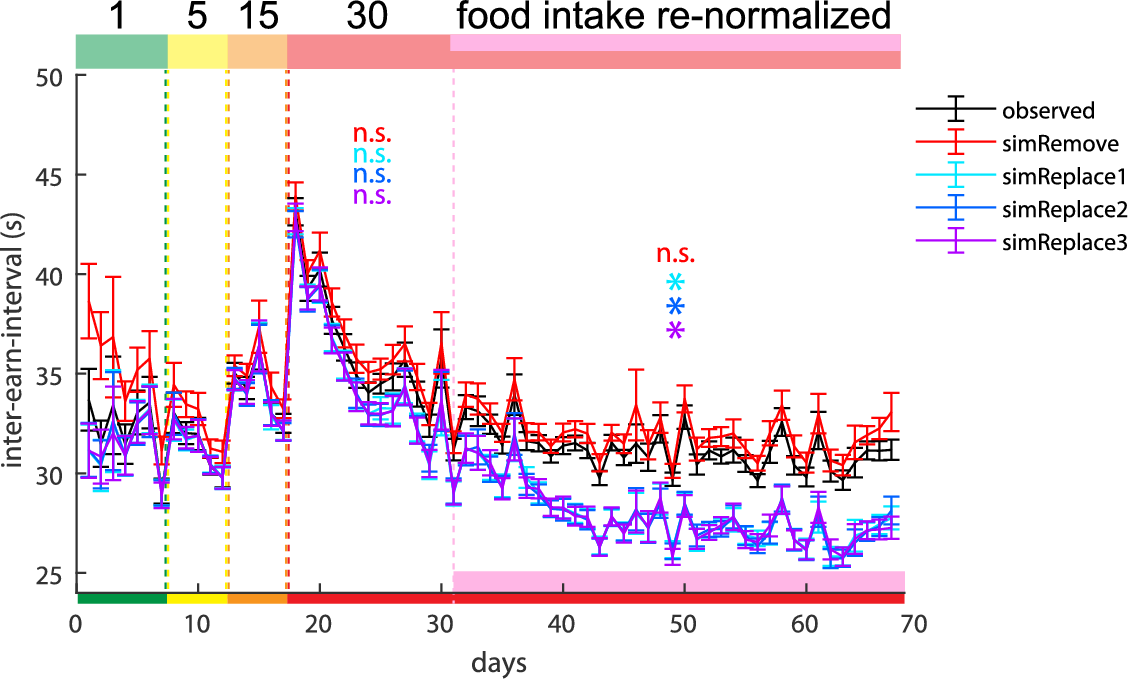

Supplement: S8 Fig — Reinforcement rate (inter-earn-interval) is plotted across days, comparing observed date (black) versus 4 different computer models that simulated what the expected reinforcement rate would be if high-VTE trials were adjusted. High- versus low-VTE trials were determined by a median split of VTE values taken across the entire experiment. The removal simulation (red) simply removed high-VTE trials before reinforcement rates were calculated. The 3 replacement simulations (cyan, blue, purple) resampled trial outcomes from low-VTE trials and differed based on how offer length was resampled when earned trials were simulated (offer length retained from the high-VTE trial, offer length randomly selected from the distribution for low-VTE trials, or offer length randomly selected from the uniform range of offers for that block, respectively). These simulations indicate no contributions to reinforcement rate due to high-VTE trials during the early 1–30 s epoch, despite having an effect late into 1–30 s training. Data presented as the cohort’s (N = 31) daily means (±1 SE). Color code on the x-axis reflects the stages of training (offer cost ranges denoted from 1 to the number on the top of the plot). * indicates significant difference compared against observed data. n.s., not significant; VTE, vicarious trial and error. (TIF) [file pbio.2005853.s008.tif]
