# Supplementary material for: Mice learn to avoid regret
Source: PLoS Biol. 2018 Jun 21;16(6):e2005853. doi: 10.1371/journal.pbio.2005853 (PMC6013153; doi:10.1371/journal.pbio.2005853)
Supplement: S1 Text — Additional analyses and discussion are available in the supplemental text, including analyses and discussion on (1) early conditioned place behaviors; (2) the development of default responses in the reward-rich components of training; (3) how demand elasticity changed across the longitudinal design; (4) evidence that the mice behaved suboptimally on this task; (5) postregret compensatory valuations; and (6) the relationship between the reluctance to skip, the development of deliberative strategies as the environment became reward-scarce, and VTE behaviors. VTE, vicarious trial and error. (DOCX) [file pbio.2005853.s009.docx]

# Supporting Information: S1 Text

## Interactions between revealed subjective valuation preferences and the development of separable decision strategies.

*Flavors were ranked from least preferred to most preferred based on total pellet earnings in each restaurant at the end of each session.*

**Early conditioned place preference behaviors.** Somewhat surprisingly, beginning on day 1 of training, we found that mice, after earning and consuming food pellets on the Restaurant Row task, often lingered at the reward site before advancing to the next trial at the next restaurant. Interestingly, rodents typically spent >50% of the entire 1hr testing session engaging in this lingering behavior (Fig 3A, *t*=14.66, *p*<0.0001). The decision to linger near the reward site rather than leave may represent a strong conditioned-place-preference-like effect [55] associated with each restaurant’s unique spatial context. This decision depletes an animal’s limited time budget and comes with the cost of impeding earning a subsequent reward since self-paced trials are interdependent.

The value of a reward can be assessed in multiple ways. Often, this is measured in an instrumental manner, as how willing a subject is to take a reward (e.g., measured in amount of resources spent, effort expended, or behavioral invigoration). These behaviors are sometimes referred to as reward-seeking, reward-taking, or “wanting” valuations [56]. On this task, post-consumption lingering behavior, where no overt reward is being sought out, appears distinct from reward-taking or “wanting” valuation, and instead may reflect more general hedonic valuations embedded in contextual Pavlovian associations. However, the two valuations produced similar ordering of restaurant preferences. Evidence for this is garnered by the fact that mice lingered longer in higher preferred restaurants (S1 Fig L, *F*=365.73 *p*<0.0001). Thus, it is worth noting that such context-associated Pavlovian valuations that promote staying and a reluctance to leave may carry added weight during more traditional reward-taking processes in offer zone enter vs. skip and wait zone stay vs. quit decisions when an overt reward is being sought out *before* a pellet is earned.

**Developing default responses in a reward-rich environment.** As mice learned the structure of the Restaurant Row task in a reward-rich environment (e.g., when all offers were 1s only), mice rapidly developed stable subjective flavor preferences (S1 Fig A-B). In doing so, mice developed rapid default decision responses upon entering the offer zone of a given restaurant that differed based only on the subjective value of each restaurant’s flavor which was signaled via visual contextual cues. Mice grew accustomed to entering and earning nearly 100% of offers in their most preferred restaurants, while skipping the majority of offers in their least preferred restaurants (S1 Fig A-D, green-yellow-orange epochs – relatively reward-rich environments). This dichotomy in behavioral responses between least and most preferred restaurants is apparent in a number of economic processes measured on the Restaurant Row task. With regard to developing restaurant-specific default decisions in reward-rich environments, offer zone reaction time grew fastest to enter in most preferred restaurants (S1 Fig G, *F*=1076.04 *p*<0.0001) and fastest to skip in least preferred restaurants (S1 Fig H, *F*=70.73 *p*<0.0001) of the course of the first 17 days of this experiment. Similarly, over the course of the first 17 days of this experiment, vicarious trial and error behavior (VTE) was lowest (most stereotyped and least indecisive) when entering in most preferred restaurants (S1 Fig I, *F*=592.95 *p*<0.0001) and when skipping in least preferred restaurants (S1 Fig J, *F*=219.93 *p*<0.0001). Taken together, default ballistic responses to either enter or skip were signaled in the offer zone of restaurants via contextual spatial cues while cost information signaled via tone pitch was ignored and essentially irrelevant in reward-rich environments (S2 Fig A-B,E-F).

**Demand elasticity in longitudinal design.** Becoming accustomed to selecting default responses in a reward-rich environment where offer cost is irrelevant can become problematic when suddenly transitioning into a novel reward-scarce environment. The significant decrease in food intake and reinforcement rate observed upon transition to the 1-30s offer block (Fig 2A-B) can be entirely explained by a loss of earnings in solely the most preferred restaurant driven by adhering to pre-cost-change decision policies (S1 Fig A, *F*=20.75 *p*<0.0001). Economic theory of demand elasticity posits that purchase behaviors for luxury items respond most robustly to increases in market prices such that individuals become less capable or willing to continue purchasing such goods if fixed with the same income [57]. Conversely, highly inelastic goods are those that do not respond as much to price changes and often reflect either essential or lesser preferred goods. Using pellets earned in each restaurant as our primary dependent variable, mice were unable to continue to afford earning most preferred pellets in a reward-scarce environment at the same rate they were previously accustomed to in reward-rich environments. Our data demonstrate demand elasticity asymmetry in mouse economic responses as a function of subjective flavor preferences following an unexpected price change. Demand elasticity is well-studied in human microeconomics, however, animal neuroeconomics has only recently started to explore the decision-making phenomena underlying demand elasticity. A recent neuroeconomics study in rats on a different task demonstrated similar aspects of demand theory of elasticity where budget constraints (either compensated or not) interact with subjective flavor preferences when reward prices escalated [57]. Here, in the present study, because mice were allotted the same 1hr fixed time budget (uncompensated) across the transition from reward-rich to reward-scarce environments and were tasked with earning their only source of food for the day interdependent across days, mice consequently suffered an initial loss in food intake. This was largely due to adhering to previously-learned default decision policies in each restaurant that become insufficient in a reward-scarce environment and are thus apt to produce poor yield. As a result, mice were pressured to augment decision strategies over subsequent days / weeks. A major advantage in the present study is the longitudinal nature of this neuroeconomic task. To date, no animal neuroeconomic studies have reported a longitudinal account of how subjective value-driven demand elasticity theory manifests over the course of developing pre-price-change behaviors, immediate negative consequences of price change, and intermediate-to-long-term strategy changes that learn to adapt in a self-paced manner working with a fixed, uncompensated time budget.

**On sub-optimality.** Consistent with previous reports in rats on other neuroeconomic foraging tasks [58-59], we found here that mice behaved largely sub-optimally in this variant of the Restaurant Row task. First, it should be worth mentioning that it can be argued that the fact that mice would be willing to work differently for various flavored pellets of the same caloric value at the expense of not maximizing total food intake is overtly sub-optimal in and of itself. This aside, taking into account idiosyncratic differences in subjective flavor preferences, we wanted to characterize how mice went about making economic decisions using their limited time budget normalized to subjective flavor preferences without making assumptions about flavor value. That is, mice engaged in a number of economic decision processes that detracted from their total 1hr session time budget in ways that appeared wasteful and effectively reduced maximum potential earnings, even after taking into account their subjective flavor preferences.

We generated a computer-model that simulated Restaurant Row sessions based on each animal’s observed behaviors in order to calculate maximal predicted pellets a mouse could earn in each given restaurant on a given day. We used each animal’s daily wait zone thresholds for each restaurant to base the model around individual differences in subjective value. However, we instructed the model to minimize time-expenditure inefficiencies and used each animal’s best daily behavioral capabilities to predict maximum yield (i.e., minimal offer zone reaction time, no quits, minimal consumption and lingering time, and minimal between-restaurant travel time). Because differences on most of these metrics exist along the least-to-most preferred flavor ranking axis, we expected to find asymmetries in sub-optimal behavior across the differently ranked restaurants. We were surprised to find that mice were more sub-optimal in less preferred restaurants than in more preferred restaurants across the entire experiment (S1 Fig Q, *F*=491.22 *p*<0.0001), since mice tended to engage in more “wasteful” behaviors in higher preferred restaurants. Perhaps more telling, we found upon transitioning to the 1-30s offer block, there was a significant interaction between flavor rankings across days on sub-optimal performance (S1 Fig Q, *F*=13.57 *p*<0.0001). Mice became more sub-optimal in the most preferred restaurant immediately following the block transition while becoming gradually more optimal in less preferred restaurants (more robust changes in lesser preferred restaurants) over the subsequent two weeks. The immediate change in optimal performance in the most preferred restaurant (decreased optimality) coincided with the immediate loss in food intake mice experienced while the slower change in optimal performance in lesser preferred restaurants (increased optimality) coincided with the intermediate foraging strategy learning that took place to adapt to a reward-scarce environment and re-normalize overall food intake and reinforcement rates back to levels similar to baseline in reward-rich environments.

By no means can we conclude from these data that sub-optimality on a neuroeconomic task is intrinsically bad nor good. Rather, these data simply provide further economic insight to characterize how adhering to no-longer-sufficient decision policies disrupts reinforcement rates while driving additional learning of new foraging strategies that become more efficient over time while interacting with subjective valuation processes. Furthermore, these data make apparent that seemingly “wasteful” behaviors, such as prolonging offer zone deliberation time, which occurs in the latter portion of training (pink epoch), not only affords no changes in overall food intake or reinforcement rate but also affords no changes in optimality (S1 Fig Q, *F*=0.05 *p*=0.82), in fact even misses out on increased reinforcement rates (S8 Fig). Thus, it is clear that seemingly sub-optimal behaviors that are not directly tied to investing time in the current reward offer (i.e., prolonged deliberation to skip), may be useful in other regards, for instance, in spending time planning to avoid experiencing regret.

**A reluctance to quit in the wait zone of preferred restaurants leads to a delay in learning efficient foraging strategies in a reward-scarce environment.** Calculating time spent in the wait zone before quitting across blocks of training reveal mice generally quit relatively quickly regardless of offer cost (S3 Fig A-D). Following the transition to the 1-30s offer block, we found that mice took significantly longer to decide to quit in the wait zone in more preferred restaurants (S1 Fig K, S3 Fig E-H, *F*=200.94, *p*<0.0001). These data suggest that mice were more reluctant to change their minds before quitting more preferred offers, demonstrating an aversion to leave those restaurants. As mice learned to forage more economically efficiently in the wait zone during the 2wk adaptation period re-normalizing food intake and reinforcement rates, the economic efficiency of quit decisions can be examined by comparing time spent before quitting against time remaining in the countdown at the moment of quitting, both relative to wait zone thresholds (S3 Fig I-L). Wait zone inefficiencies depicted in Fig 4B following the transition to 1-30s offers are reflected in S3 Fig K (upper left quadrant). Interestingly, we found that mice were more inefficient in more preferred restaurants and showed more difficulty learning to forage efficiently in more preferred restaurants (S1 Fig P, *F*=3.23, *p*<0.05). This suggests that the condition-place-preference-like effect described previously in post-earn lingering time (where no overt reward was being sought), could be related to the effect during wait zone countdowns showing an aversion to leave a reward-associated context, where the higher value of more preferred restaurants strongly opposed a learning processes to adapt economically advantageous foraging strategies.

**A reluctance to skip in the offer zone of preferred restaurants leads to a delay in learning efficient deliberative strategies in a reward-scarce environment.** We also found interesting interactions between context-associated valuation biases in more preferred restaurants that hindered long-term learning processes observed in the offer zone. In the 1-30s offer block, mice learned to oppose their default decision-making strategies learned in reward-rich environments, i.e., accepting nearly every offer in the offer zone of higher preferred restaurants. In doing so, they displayed significantly slower reaction times (S1 Fig H, *F*=88.78, *p*<0.0001) and increased vicarious trial and error behavior (VTE, S1 Fig J, *F*=38.74, *p*<0.0001) when skipping in the offer zone of more preferred restaurants. This suggests that mice again demonstrated an aversion to leave the offer zone of the current restaurant that scaled with subjective flavor preference, in the offer zone, much like quitting in the wait zone and lingering near the reward site post-consumption. The fact that skip decisions and not enter decisions displayed increased VTE and more pause-and-reorient behaviors in a reward-scarce environment interrupting initial default “almost-enter” decisions suggests that skip decisions recruited an additional delayed process that learned to break initial ballistic snap-judgements and ultimately make the economically advantageous decision to skip expensive offers.

We characterized how mice deliberated in the offer zone as a function of offer value (wait zone threshold minus offer cost) over the course of training in the 1-30s offer block (S4 Fig A-B). We found that, over prolonged training, offer zone reaction time and VTE behavior gradually increased and took on an inverted-U shape that grew stronger with more training, centered near 0 valued offers, but shifted toward negatively valued offers. These are expensive offers that, early in training, would have been entered-then-quit, that late in training, were deliberated upon and ultimately skipped. This inverted-U shape deliberative behavior was present regardless of flavor preference (S4 Fig C-D).

When characterizing the economic efficiency of offer zone decisions over time, we found that mice were more inefficient in more preferred restaurants, struggling longest in the most preferred restaurant through to the end of the experiment (S1 Fig O, *F*=20.72, *p*<0.0001). We adopted a signal detection theory approach to further characterize the development of and biases in value-based discriminability over the course of prolonged training (S5 Fig). We found that receiver operator characteristic (R.O.C.) curves could be used to capture deliberative learning strategies that took place in the offer zone. The area under the R.O.C. curves increased in all restaurants during the 1-30s offer block indicating mice learned to become better value-based signal detectors in the offer zone (S5 Fig C, *F*=512.84, *p*<0.0001). R.O.C. skew captured value-based discriminability error biases in the offer zone (asymmetry towards errors of accepting high cost offers as opposed to rejecting low cost offers) that interacted with subjective flavor preferences (more skew in more preferred restaurants), that did not begin to improve until the pink epoch, less so in higher preferred restaurants (S5 Fig D, *F*=12.94, *p*<0.0001).

**Post-regret compensatory valuations.** We found interactions between subjective flavor preferences in how mice behaved in subsequent restaurants following enter-then-quit decisions compared to skip decisions. That is, following a quit decision, if mice entered their least preferred restaurant next, they demonstrated different immediate post-regret compensatory valuations than if they entered their most preferred restaurant next.

First, mice were more likely to make more rapid decisions in subsequent restaurants following quits regardless if the next restaurant was their least or most preferred flavor (S6 Fig B, *F*=37.31, *p*<0.0001; post-hoc Tukey, least: *t*=5.39, *p*<0.0001; most: *t*=3.28, *p*<0.01). This suggests mice were more likely to make snap judgements overall following quit-induced regret. Interestingly however, mice were only more likely to enter offers following quits if the next restaurant was their least preferred flavor (S6 Fig B, *F*=55.50, *p*<0.0001; post-hoc Tukey, least: *t*=9.83, *p*<0.0001; most: *t*=0.81, *p*=0.85). Conversely, mice were only more likely to linger less after consuming earned pellets in the subsequent restaurant if the next restaurant was their most preferred flavor (S6 Fig B, *F*=5.10, *p*<0.05; post-hoc Tukey, least: *t*=0.89, *p*=0.81; most: *t*=3.37, *p*<0.05). These data suggest that the ways in which mice “made up for lost efforts” and immediately compensated for regret manifested differently based on the subjective value of the flavor of the subsequent restaurant.

Mice were more willing to accept offers in restaurants they typically defaulted to reject following regret – a finding that may not be observable in most preferred restaurants due to a possible ceiling effect. Mice were also quicker to consume earned pellets and leave the reward site in restaurants they typically lingered at for an extended period of time – a finding that may not be observable in least preferred restaurants due to a possible floor effect. Taken together, these data provide interesting insight when considering how the immediate effects of regret on subsequent decision-making may foster more liberal vs. conservative economic responses, different responses of which may depend on subjective valuation processes of subsequent reward opportunities.

**Controlling for the effects of early Vicarious Trial and Error changes in offer-zone behaviors on reinforcement rate renormalization.** Because skip VTE behaviors begin to increase early in 1-30s training during the time period when food intake and reinforcement is re-normalizing, we wanted to test if any offer-zone behaviors could be contributing to this re-normalization. First, it is worth nothing that skip behaviors are relatively rare events during this epoch of time. Second, choice outcomes in the offer zone during this epoch remain stable and elevated, as mice are apt to accept most offers regardless of cost. During this epoch, decreases in quit time can explain decreases in reinforcement rate. Therefore, at first glance, increases in skip time would only oppose a decrease in reinforcement rate. Nonetheless, we wanted to test the role of high VTE events in influencing reinforcement rate more robustly and directly. We ran computer models to predict how reinforcement rate would be altered if high VTE trials were corrected for. To do this, we first determined high vs. low VTE by taking a median split of IdPhi distributions across the entire experiment. We generated 4 types of simulations and compared predicted reinforcement rate against the observed reinforcement rate (S8 Fig). We either entirely removed or replaced high VTE trials with estimated outcomes selected low VTE trials and found no changes from observed reinforcement rate during the early 1 day -30s epoch (*F*=0.08, *p*=0.77).

Interestingly, we found effects of high VTE replacement with low VTE estimates compared to observed reinforcements rates and high VTE removal simulations late in 1-30s training. Reinforcement rate was significantly higher (lower inter-earn interval) in all three replacement simulation variants compared to observed data and predicted data from high VTE removal simulations (*F*=11.98, *p*<0.0001). Furthermore, reinforcement rates in the high VTE replacement simulations were significantly higher (lower inter-earn interval) than average reinforcement rates in relatively reward-rich environments only late in training (pink epoch vs. first three training blocks, *F*=33.96, *p*<0.0001). Taken together, this suggests that reinforcement rate *could* theoretically improve even better than observed performance only late in training. Thus, when deliberative strategies in the offer zone resulted in changes in offer zone thresholds (i.e., changes in choice outcomes depending on the cost of the offer), it would appear that these decisions were made despite missing out on potentially “better than observed” and even “better-than-before” reinforcement rates. Importantly, S8 Fig reveals that the excess VTE late in training does, in fact, decrease the reward receipt rate even further below what could, theoretically, be achieved. This implies that there must be a “hidden utility” to deliberating or a “hidden cost” to not [58]. We argue that this utility is rooted in the subjective hidden cost of regret, thus producing a utility benefit to regret avoidance. Again, this would suggest that the decision strategy changes observed in the late 1-30s training are driven by a separate process distinct from that in early 1-30s training, with the latter being VTE-dependent.

55. Clark, J., Hollon, N., Phillips, P. Pavlovian valuation systems in learning and decision making. *Current opinion in neurobiology* 22, 1054–1061 (2012).

56. Robinson, T.E., Berridge, K.C. The neural basis of drug craving: an incentive-sensitization theory of addiction. *Brain research. Brain research reviews* 18, 247–91 (1993).

57. Van Wingerden, M,, Marx, C., Kalenscher, T. Budget Constraints Affect Male Rats’ Choices between Differently Priced Commodities. *PloS One* 1-20 (2015).

58. Wikenheiser, A.M., Stephens, D.W., Redish, A.D. Subjective costs drive overly patient foraging strategies in rats on an intertemporal foraging task. *PNAS* 110, 8308–13 (2013).

59. Carter, E.C., Redish, A.D. Rats value time differently on equivalent foraging and delay-discounting tasks. *J Exp Psychol Gen* 145, 1093–101 (2016).

60. Sweis, B.M., Abram, S., Schmidt, B., Breton, Y., Thomas, M.J., MacDonald, A.W., Redish, A.D. Sunk cost effects appear similar in parallel neuroeconomic foraging tasks in mice, rats, and humans. *Society for Neuroeconomics,* Toronto, Canada (2017).
